# rVSVΔG-ZEBOV-GP Vaccine Is Highly Immunogenic and Efficacious Across a Wide Dose Range in a Nonhuman Primate EBOV Challenge Model

**DOI:** 10.3390/v17030341

**Published:** 2025-02-28

**Authors:** Amy C. Shurtleff, John C. Trefry, Sheri Dubey, Melek M. E. Sunay, Kenneth Liu, Ziqiang Chen, Michael Eichberg, Peter M. Silvera, Steve A. Kwilas, Jay W. Hooper, Shannon Martin, Jakub K. Simon, Beth-Ann G. Coller, Thomas P. Monath

**Affiliations:** 1 US Army Medical Research Institute of Infectious Diseases (USAMRIID), Porter Street, Fort Detrick, Frederick, MD 21702, USA; amy.shurtleff@cepi.net (A.C.S.); john.c.trefry.civ@mail.mil (J.C.T.); mmesunay@gmail.com (M.M.E.S.); peter.silvera@ablinc.com (P.M.S.); steven.a.kwilas.civ@health.mil (S.A.K.); jay.w.hooper.civ@health.mil (J.W.H.); shannon.s.martin2.civ@mail.mil (S.M.); 2 Merck & Co., Inc., 126 E. Lincoln Ave., Rahway, NJ 07065, USA; kenneth_liu@merck.com (K.L.); ziqiang.chen@merck.com (Z.C.); michael.eichberg@merck.com (M.E.); jakub.simon@orelconsulting.com (J.K.S.); bethann.coller@gmail.com (B.-A.G.C.); 3 NewLink Genetics Corp., Loop Dr #5100, Ames, IA 50010, USA; tom@quigleybio.com

**Keywords:** Ebolavirus, ERVEBO, vaccine, nonhuman primates, vesicular stomatitis virus

## Abstract

The recombinant vesicular stomatitis virus-Zaire Ebolavirus envelope glycoprotein vaccine (rVSVΔG-ZEBOV-GP) was highly effective against Ebola virus disease in a ring vaccination trial conducted during the 2014–2016 outbreak in Guinea and is licensed by regulatory agencies including US FDA, EMA, and prequalified by WHO. Vaccination studies in a nonhuman primate (NHP) model guided initial dose selection for clinical trial evaluation. We summarize two dose-ranging studies with the clinical-grade rVSVΔG-ZEBOV-GP vaccine candidate to assess the impact of dose level on immune responses and efficacy in an NHP Ebola virus (EBOV) challenge model. Forty-six cynomolgus macaques were vaccinated with a wide range of rVSVΔG-ZEBOV-GP doses and challenged 42 days later intramuscularly with 1000 pfu EBOV. Vaccination with rVSVΔG-ZEBOV-GP induced relatively high levels of EBOV-specific IgG and neutralizing antibodies, measured using the same validated assays as used in rVSVΔG-ZEBOV-GP clinical trials. Similar responses were observed across dose groups from 1 × 10^8^ to 1 × 10^2^ pfu. A single vaccination conferred 98% protection from lethal intramuscular EBOV challenge across all dose groups. These results demonstrate that robust antibody titers are induced in NHPs across a wide range of rVSVΔG-ZEBOV-GP vaccine doses, correlating with high levels of protection against death from EBOV challenge.

## 1. Introduction

Ebolaviruses are members of the *Filoviridae* family, a group of filamentous, enveloped ribonucleic acid (RNA) viruses, maintained in nature in an enzootic cycle most likely involving fruit bats [1] and in humans through persistent infection [2,3,4]. After introduction from the natural cycle, the virus is transmitted by person-to-person contact. The Ebola virus (EBOV), *Orthoebolavirus zairense*, formerly known as *Zaire ebolavirus*, has been responsible for the vast majority of human cases of Ebola virus disease (EVD), including the 2014–2016 outbreak in West Africa and multiple subsequent outbreaks, including those in the Democratic Republic of the Congo (2018–2022), Uganda (2022), and Guinea (2021) [5]. Initially, patients with EVD exhibit nonspecific symptoms, but severe disease can quickly develop profuse diarrhea, hemorrhagic symptoms, and multi-organ dysfunction, often with high mortality [6]. In a meta-analysis that included 20 outbreaks from 1976 to 2014 as recorded by the World Health Organization (WHO), the case fatality rate for EBOV was 76% [7]. Two monoclonal antibody (mAb) treatments (Inmazeb and Ebanga) were found to reduce EVD-associated mortality and have been approved by the US Food and Drug Administration (FDA) since 2020 [8,9,10]. At the time of the 2014–2016 outbreak, there was no approved vaccine or treatment available for EVD.

rVSVΔG-ZEBOV-GP is a live attenuated recombinant viral vaccine in which the gene encoding the vesicular stomatitis virus (VSV) glycoprotein (GP) G is replaced with the GP gene of EBOV [11]. Eight Phase 1 [12,13,14,15,16,17,18,19] and five Phase 2/3 [20,21,22,23,24] clinical trials of rVSVΔG-ZEBOV-GP were conducted during the 2014–2016 outbreak with more than 18,000 individuals receiving at least one dose of rVSVΔG-ZEBOV-GP [11]. In some cases, long-term follow-up of participants, up to 5 years post-vaccination, extended beyond the outbreak [25]. The vaccine was generally well tolerated in healthy participants [11,12,13,14,15,16,17,18,19,20,21,22,23,24,26]. A pivotal Phase 3 study conducted in Guinea using a ring vaccination protocol demonstrated 100% efficacy in individuals ≥10 days after receiving a single vaccination of rVSVΔG-ZEBOV-GP at 2 × 10^7^ pfu [22]. Efficacy and safety data from these trials formed key components in licensure submissions to Regulatory Agencies including the FDA and European Medicines Agency (EMA). The rVSVΔG-ZEBOV-GP vaccine (ERVEBO^®^; also known as V920; Merck & Co., Inc., Rahway, NJ, USA) received conditional (November 2019) and full market authorization (January 2021) from the EMA for adults aged ≥18 years to protect against EVD caused by EBOV. The EMA approved an expanded indication for ERVEBO in September 2023 to include individuals aged ≥1 year [27]. In December 2019, the US FDA authorized the vaccine for adults aged ≥18 years [28], and in August 2023, they expanded its indication to include individuals aged ≥12 months [29]. As of December 2023, rVSVΔG-ZEBOV-GP has also been approved in the UK, Canada, Switzerland, and 11 countries in Africa (Burundi, Central African Republic, Cote d’Ivoire, Democratic Republic of the Congo, Ghana, Guinea, The Republic of the Congo, Rwanda, Sierra Leone, Uganda, and Zambia) [30]. Since approval of the rVSVΔG-ZEBOV-GP vaccine, an additional Ebola vaccine has been approved by the EMA but requires two doses for primary immunization [31].

Preclinical studies conducted prior to the 2014–2016 EBOV outbreak consistently demonstrated the efficacy of rVSVΔG-based vaccines in protecting nonhuman primates (NHPs) against lethal Ebola infections [32,33,34,35]. These studies evaluated research-grade preparations of the vaccine at doses of approximately 1 × 10^7^ pfu, which were 100% effective in preventing Ebolavirus disease in NHPs when a single dose was delivered about 28 days prior to EBOV challenge. Other preclinical studies demonstrated a critical role for antibodies in the vaccine-induced protection [36] and demonstrated that the rVSVΔG-ZEBOV-GP vaccine was well tolerated in healthy NHP as well as in simian–human immunodeficiency virus (SHIV)-infected NHPs [32]. Collectively, these preclinical data contributed to the decision to advance the vaccine candidate into Phase 1 clinical trials in late 2014.

In parallel with ongoing Phase 1 and Phase 2/3 clinical trials of rVSVDG-ZEBOV-GP in 2014–2015, the US Department of Defense, in collaboration with Merck & Co., Inc. (Rahway, NJ, USA), conducted two dose-ranging NHP studies with clinical-grade preparations of rVSVΔG-ZEBOV-GP to evaluate immunogenicity and efficacy of this vaccine at doses similar to those used in the clinical studies. Across these two NHP studies, animals were vaccinated with a wide range of rVSVΔG-ZEBOV-GP doses (1 × 10^8^ to 3 × 10^2^ pfu) with the goal of inducing a range of immune responses, which were used in Study 1 to support dose selection for clinical studies and were used in Study 2 to inform correlates of protection analyses for potential immunobridging between NHP and human responses and efficacy. Ebolavirus envelope glycoprotein (EBOV-GP)-specific antibody responses were assessed using the same validated enzyme linked immunosorbent assay (ELISA) and 60% plaque reduction neutralization test (PRNT_60_) methods used in clinical studies. This approach was used to help better align across species for immunobridging evaluations and to estimate efficacy of filovirus vaccines in humans in situations where studies to demonstrate protection against disease may not be feasible.

Here, we report results from both studies, which, together with clinical safety and efficacy data and additional nonclinical toxicology data, supported the application for regulatory approval and product licensure and served as the foundation for understanding immune responses and protection in a relevant animal model.

## 2. Materials and Methods

### 2.1. Study Design

An overview of the study design of the two immunogenicity and efficacy studies of rVSVΔG-ZEBOV-GP in NHPs, including the dose regimen of vaccination, is shown in Table 1. Both studies were conducted by the United States Army Medical Research Institute of Infectious Diseases (USAMRIID).

Development of the rVSVΔG-ZEBOV-GP vaccine has been thoroughly reviewed, and the product has been described previously [11]. A clinical-grade rVSVΔG-ZEBOV-GP vaccine candidate manufactured by IDT Biologika (lot # 003 05 13 in Study 1 and lot # 001 10 14 in Study 2) was used here. EBOV (strain Kikwit) was isolated from a human case in the Democratic Republic of the Congo in 1995 (USAMRIID challenge stock “R4415”) and is the most frequently used EBOV isolate for NHP studies [37,38].

### 2.2. Animals

In Study 1, 27 adult cynomolgus macaques (*Macaca fascicularis*) of Asian origin (age range, 4–7 years; weight range, 3.7–10.6 kg; 13 males, 14 females) were randomized into one of four groups, eight monkeys per group in three experimental groups and three monkeys in a control group. Animals in the three experimental groups were vaccinated by intramuscular (IM) injection (in the deltoid muscle) of 1 × 10^8^, 2 × 10^7^, or 3 × 10^6^ plaque-forming units (pfu) of rVSVΔG-ZEBOV-GP. In Study 2, 24 adult cynomolgus macaques (*Macaca fascicularis*) of Cambodian origin (age range, 4–11 years; weight range, 3.3–7.8 kg; 11 males, 13 females) were randomized into one of six groups, four or five monkeys per group in five experimental groups and two monkeys in a control group. Animals in the experimental groups were vaccinated by IM injection (in the deltoid muscle) of 3 × 10^6^, 3 × 10^5^, 3 × 10^4^, 3 × 10^3^, or 3 × 10^2^ pfu of rVSVΔG-ZEBOV-GP. The control groups in both studies received diluent/saline only. In both studies, study personnel were blinded to the animal group assignments. Forty-two days after vaccination, all animals were challenged with EBOV at a target dose of 1000 pfu, which was administered intramuscularly in 0.5 mL (Study 2) or 1 mL (Study 1) to the right quadriceps of anesthetized animals. Previous studies have shown this dose of EBOV to be uniformly lethal within 5–8 days after challenge [39]. Prepared challenge inoculum was titrated for dose verification using a validated assay [40].

The animals were observed at least once daily, with more frequent observations on days when at least one animal exhibited clinical signs of disease. Physical examinations under anesthesia including body weight, temperature, and physical examination observations (e.g., observations of vaccination or challenge injection site, excreta, presence of petechial rash, and lymphadenopathy by palpation) were conducted 4 days prior to the vaccination dose, during the vaccination phase on Days −4, 0, 1–4 (in Study 2 only), 7, 14, 28, and 35 (Study 2) or 36 (Study 1), and during the challenge phase on Days 0, 3, 5, 7, 10, 14, 21, and either at end of the in-life phase (Days 28–31) and/or at termination (Study 1) or on Day 28 (Study 2). In addition, blood was collected for analysis of hematology and clinical chemistry on Days −4, 0, 1–4 (for Study 2 only), and 7 of the vaccination phase and Days 0, 3, 5, 7, 10, 14, 21, and 28 of the challenge phase (including coagulation parameters for Study 2).

### 2.3. Responsiveness Scores

During the vaccination phase (Days 0–10), animals were assigned a responsiveness score from 0 to 3: 0 = active; 1 = decreased activity; 2 = mildly unresponsive (becomes active when approached), occasional prostration; and 3 = moderate unresponsiveness (requires prodding), demonstrates weakness. During Days 42–56 (Days 0–14 of the challenge phase), animals were assigned a responsiveness score from 0 to 5: 0 = active; 1=decreased activity; 2 = mildly unresponsive (becomes active when approached), occasional prostration; 3 = moderate unresponsiveness (may require prodding to respond), weakness; 4 = moderate to severe unresponsiveness requires prodding, moderate prostration; and 5 = moribund, severe unresponsiveness, pronounced prostration. After Day 56, the number of observations was reduced to twice daily, with the second observation performed at least 6 h later.

### 2.4. Humoral Immune Response

Antibody responses to the rVSVΔG-ZEBOV-GP vaccine were measured in serum samples collected during the vaccination phase. Anti-EBOV GP immunoglobulin G (IgG) titers were determined using the validated ZEBOV-GP IgG ELISA conducted at Q^2^ Solutions (San Juan Capistrano, CA, USA) as previously described [17,41]. Briefly, serum samples were serially diluted and added to microtiter plates that had been coated with purified recombinant ZEBOV-GP (Kikwit strain) overnight. After incubation, plates were washed prior to the addition of a horseradish peroxidase-conjugated goat anti-human IgG secondary antibody. Plates were washed again and 3,3′,5,5′–tetramethylbenzidine (TMB) substrate was added. The enzymatic reaction was stopped with a sulfuric acid solution and optical density (OD) was measured on an ELISA plate reader. Anti-GP IgG concentrations were calculated from a dilution series of a reference standard consisting of pooled human sera using a 4-parameter logistic (4PL) curve fit. GP-ELISA antibody titers were reported in GP-ELISA units per milliliter (EU)/mL. The assay was validated for the analysis of both human and NHP samples based on in-depth parallel assessments [42] and was demonstrated to have a lower limit of quantitation (LLOQ) of 13.62 EU/mL at Q^2^ Solutions. Seroresponse for GP-ELISA was assessed in two ways: a 2× increase from baseline to 200 EU/mL or higher or a four-fold increase from baseline [43]. A post-challenge ELISA was conducted in Study 2 under level 4 biocontainment at USAMRIID using a non-validated ELISA (Supplementary Methods). Prior to challenge, assessment of virus-neutralizing antibody titer on Days −4, 0 (in Study 2 only), 7, 14, 28, and 35 (Study 2) or 36 (Study 1) during the vaccination phase was also measured at biosafety level (BSL)-2 in a validated assay based on neutralization of the vaccine virus as previously described [17]. In brief, serially diluted serum samples were mixed with rVSVΔG-ZEBOV-GP and incubated for 20 h to allow neutralization prior to adding to Vero cells. A methylcellulose overlay was added an hour later, and infected cells were incubated 2 days before plaques were visualized using a crystal violet stain and counted. Determination of the 60% neutralizing titer (PRNT_60_) is based upon the percent reduction in viral plaques in the presence of serum compared to that of the virus control without serum and is calculated by linear regression. This vaccine virus-based PRNT was validated for the analysis of both human and NHP samples and was conducted at Q^2^ Solutions. The PRNT_60_ lower limit of detection is 20 and LLOQ is 35. For collection of interim data for informational purposes, pre-challenge anti-EBOV GP IgG titers and pseudovirion neutralization assay (PsVNA) [19] were first determined in both studies at USAMRIID using non-validated research-grade methods (Supplementary Methods).

### 2.5. rVSVΔG-ZEBOV-GP Viremia and EBOV RNA

In Study 2, VSV-ZEBOV vaccine virus plasma levels post-vaccination were measured at Q^2^ Solutions using a quantitative RT-PCR (qRT-PCR) assay as previously described [17]. This assay is qualified for testing human plasma samples but is not species specific, therefore assay performance is expected to be similar with NHP plasma samples. For the assay method used at the time of these NHP studies, the lower limit of detection was 62.5 copies/mL, and LLOQ was 156.25 copies/mL.

Wild-type EBOV RNA in plasma post-challenge was measured at USAMRIID for both studies using a validated qRT-PCR method as previously described [38].

### 2.6. Hematology and Clinical Chemistry

Hematology parameters were determined from blood samples collected in tubes containing EDTA using the ADVIA 120 Hematology System (Siemens Healthineers, Erlangen, Germany). Serum chemistry analytes were measured using a Piccolo Xpress analyzer (Abaxis, Union City, CA, USA) (Study 1) or a Vitros^®^ 350 Chemistry System (Ortho Clinical Diagnostics, Raritan, NJ, USA) (Study 2). In Study 2, coagulation parameters were analyzed using the Sysmex CA-1500 instrument (Siemens Healthineers, Erlangen, Germany).

### 2.7. Anatomical Pathology

Upon death or euthanasia, necropsies were conducted under BSL-4 containment. Collected tissues for histopathology were fixed in 10% neutral buffered formalin. After a minimum of 21 days’ formalin fixation, the tissue samples were trimmed, routinely processed, embedded in paraffin, and sections cut at 5 µm for histology. The histology slides were deparaffinized and stained with hematoxylin and eosin. In addition, immunohistochemistry for detection of EBOV antigen post-challenge was performed on collected tissues including samples of the lung, liver, spleen, kidney, and inguinal lymph nodes using an Envision-PO kit (Agilent, Santa Clara, CA, USA). For immunohistochemistry analysis, a mouse monoclonal anti-EBOV antibody (USAMRIID #702/703) [44] was used at a dilution of 1:8000. After deparaffinization and peroxidase blocking, sections were covered with primary antibody and incubated at room temperature for 30 min. The sections were rinsed, secondary anti-mouse IgG antibody (peroxidase-labeled polymer) applied for 30 min, rinsed, substrate–chromogen solution applied for 5 min, rinsed, and then stained with hematoxylin.

In Study 2, in situ hybridization (ISH) was performed on the eye tissue of all animals using the ViewRNA^TM^ ISH Tissue Assay kit (ThermoFisher Scientific, Waltham, MA, USA). For this assay, 20 ZZ probes (18–25 base-long oligonucleotide pairs complementary to target RNA) against EBOV NP were designed and synthesized. After the deparaffinization and peroxidase blocking process, eye sections were covered with ISH probes and incubated at 40 °C in a hybridization oven for 2 h, rinsed, the ISH signal was amplified by applying Pre-amplifier and Amplifier conjugated with alkaline phosphatase, and then a fast red substrate–chromogen solution was applied for 10 min at room temperature. Slides were counterstained with hematoxylin and evaluated by a veterinary pathologist.

### 2.8. Statistical Analysis

For both studies, the primary analyses estimated geometric mean titers (GMTs) of the anti-EBOV GP IgG using the validated ZEBOV-rGP ELISA and the EBOV GP-specific neutralizing antibody titers using the validated rVSVΔG-ZEBOV-GP PRNT_60_ across all treatment groups on all study days tested. GMT estimates, standard deviations (SDs), and 95% confidence intervals (CIs) were based on an analysis of variance (ANOVA) model including treatment group as a categorical covariate. The ANOVA model was performed separately on all study days tested. Other analyses included estimation of geometric mean ratios (GMRs) of ZEBOV-rGP ELISA and rVSVΔG-ZEBOV-GP PRNT_60_ between all treatment groups compared pairwise, with GMR estimates and 95% CIs based on the same ANOVA model performed separately on all study days. A test of linear trend for vaccine dose was performed by both including the placebo group and excluding the placebo group by day using the SAS (Version 9.4) CONTRAST statement, with *p* values based on the same ANOVA model. Since these were both estimation studies, no multiplicity adjustments were made.

An integrated analysis of Study 1 and Study 2 was performed. The relationships between survival and dose or between survival and titer response were assessed using a Cox proportional hazards model and logistic regression, where survival was the outcome and dose or titer response was the predictor. Survival analyses were performed using SAS Version 9.4.

A post hoc analysis was conducted to evaluate a predictive threshold of immune response above which animals are protected. An overall vaccine group was formed by combining all NHP in non-zero vaccine dose groups (*N* = 45) and a placebo control group composed of all NHP in zero dose groups (*N* = 5). The data from the same assay on the same relative study day were pooled across the two studies by dose group. A logistic regression model was used to model the risk of death from EBOV infection in NHP animals as a continuous function of immune response.

## 3. Results

### 3.1. Observations After Vaccination

In both studies, no signs of illness or Draize reaction were observed as a result of vaccination. In Study 1, most animals gained weight after vaccination and most had a slight decrease in body temperature noted on Day 7, although body temperatures commonly fluctuated. There were no clear hematology or clinical chemistry changes. On Day 28, one animal in the 2 × 10^7^ pfu group (Study 1) had low body temperature and a distended abdomen. The animal’s condition rapidly deteriorated despite repeated treatment to relieve gas in the stomach, and it was ultimately euthanized on Day 28 due to gastric bloat as determined by a veterinarian pathologist [45]. In Study 2, the average body weight was stable, there was no observed pattern of a febrile reaction, and no remarkable hematology or clinical chemistry changes.

### 3.2. Antibody Response After Vaccination

**Figure 1:**
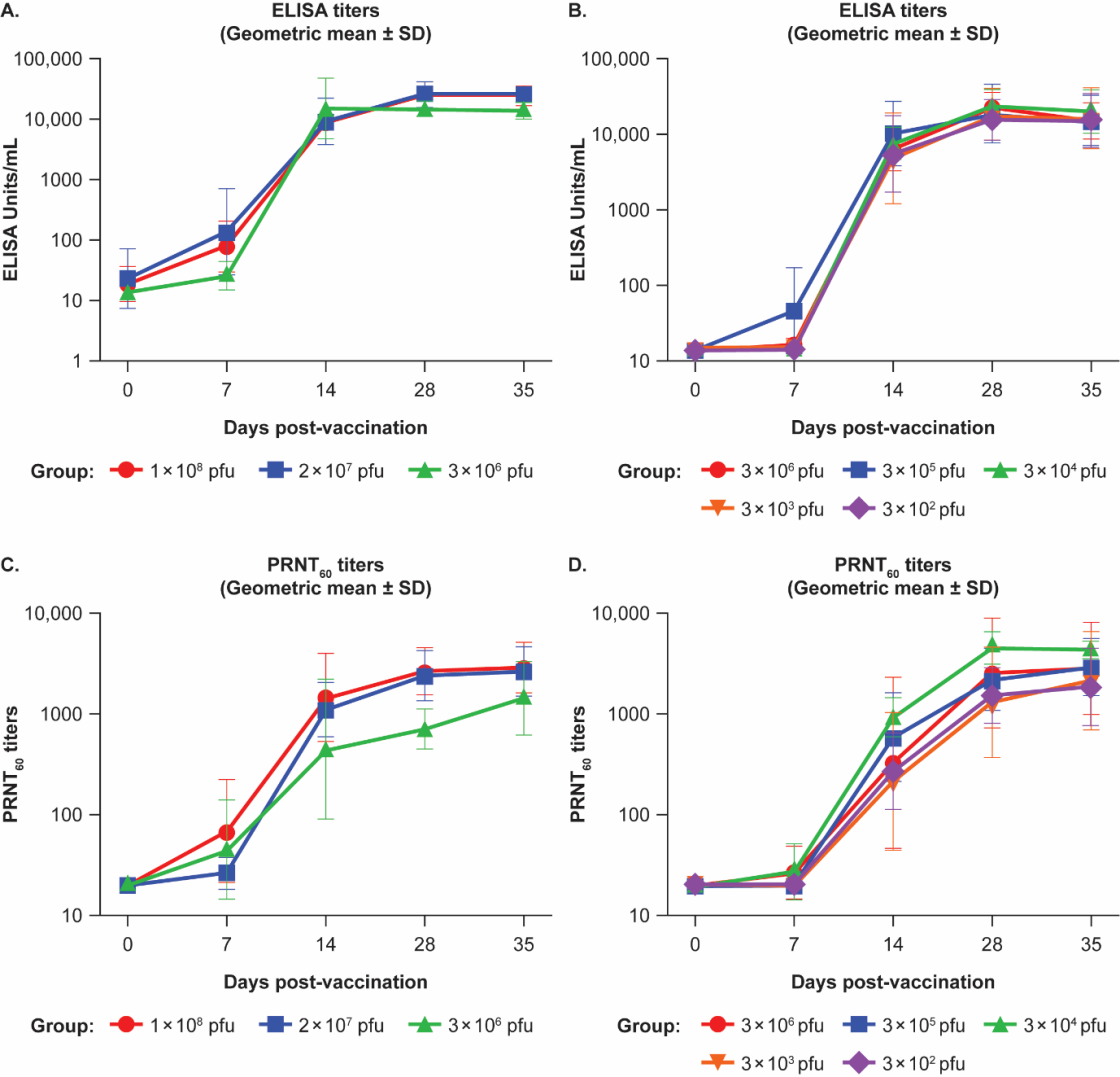
Antibody responses in nonhuman primates after single intramuscular administration of various doses of rVSVΔG-ZEBOV-GP determined using validated assays. Ebolavirus-glycoprotein (EBOV-GP)-specific immunoglobulin G antibody titers in (**A**) Study 1 and (**B**) Study 2. 60% plaque-reduction neutralization test (PRNT_60_) titers in (**C**) Study 1 and (**D**) Study 2. Three experimental groups (eight monkeys per group) in Study 1 received 1 × 10^8^, 2 × 10^7^, or 3 × 10^6^ plaque-forming unit (pfu) intramuscular (IM) doses of rVSVΔG-ZEBOV-GP. Five experimental groups (four or five monkeys per group) in Study 2 received 3 × 10^6^, 3 × 10^5^, 3 × 10^4^, 3 × 10^3^, or 3 × 10^2^ pfu IM doses of rVSVΔG-ZEBOV-GP. Antibody titers are presented as geometric means. ELISA, enzyme-linked immunosorbent assay; SD, standard deviation.

Vaccinated animals at all dose levels in both studies developed robust EBOV-GP-specific IgG binding ELISA titers as measured in the validated assay, with responses detectable in some animals starting at Day 7 post-vaccination, detectable in all vaccinated animals by Day 14 and plateauing at 28 to 35 days post-vaccination (Figure 1A,B). Day 28 geometric mean ELISA responses in the vaccinated animals ranged from 15,610 to 26,097 EU/mL. A test of linear trend for vaccine dose showed a linear trend for vaccine dose on Days 7, 14, 28, and 35 (*p* < 0.0001), driven primarily by low ELISA titers for placebo animals. When placebo animals were excluded, this trend was observed only on Day 7 (*p* < 0.0001), suggesting that higher vaccine doses are associated with higher titers at early time points post-vaccination. Univariate Cox proportional hazards models performed on dose, and the ELISA titer on Day 35 showed no effect of dose (*p* = 0.3112), but higher ELISA titers on Day 35 were associated with a lower hazard ratio for death (*p* = 0.0262). Similarly, univariate logistic regression analyses performed on dose, and the ELISA titer on Day 35 showed no effect of dose (*p* = 0.2908), but higher ELISA titers on Day 35 were associated with a lower odds ratio for death (*p* = 0.0127). Of the 44 surviving NHPs (out of 45 in the vaccinated groups), the Day 28 GP-ELISA response was 4540 EU/mL or higher, which represents a 300× or higher rise over prevaccination, compared with six non-survivors (5/5 in placebo group and 1/45 in vaccinated group) where Day 28 GP-ELISA response was 14 EU/mL (1× rise) in the placebo group (*n* = 5) and 14,226 EU/mL (>1000× rise) in the vaccinated group (*n* = 1).

Importantly, at all doses tested in both studies, vaccinated animals developed robust EBOV-GP-specific neutralizing antibody titers that were measured in the validated assay, with responses detectable in some animals starting at 7 days post-vaccination, and in all vaccinated animals by Day 28 post-vaccination (Figure 1C,D). PRNT_60_ titers remained elevated, with responses plateauing at 28 to 35 days post-vaccination. Day 28 geometric mean PRNT_60_ titers in the vaccinated animals across both studies ranged from 1084 to 4566.

A test of linear trend for vaccine dose showed a linear trend for vaccine dose on Days 7, 14, 28, and 35 (*p* < 0.0049), driven primarily by low PRNT titers for placebo animals. When placebo animals were excluded, the trend was observed on Days 7 and 14 (*p* < 0.0076), suggesting that higher vaccine doses are associated with higher titers at early time points post-vaccination. Univariate Cox proportional hazards models performed on dose and the PRNT titer on Day 35 showed no effect of dose (*p* = 0.3112) or PRNT titer on Day 35 (*p* = 0.1063) for the hazard ratio for death. Similarly, univariate logistic regression analyses performed on dose and the PRNT titer on Day 35 showed no effect of dose (*p* = 0.2908) and or PRNT titer on Day 35 (*p* = 0.0624) for the odds ratio for death. Of the 43 surviving NHP with available PRNT_60_ data (44/45 survived in the vaccinated groups), the Day 28 PRNT response was 156 or higher, which represents an 8× or higher rise over pre-vaccination, compared with six non-survivors (5/5 in placebo group and 1/45 in vaccinated group) where Day 28 PRNT response was 20 (assay LLOQ), representing a 1× rise in the placebo group (*n* = 5) and 531 (27× rise) in the vaccinated group (*n* = 1).

Non-validated research-grade assays were initially used to collect interim data on anti-EBOV GP IgG titers and neutralizing antibody titers as measured by PsVNA. Results from the non-validated ELISA for both studies and results from the PsVNA assay are shown in Supplementary Figure S1.

### 3.3. rVSVΔG-ZEBOV-GP Vaccine-Associated Viremia

**Figure 2:**
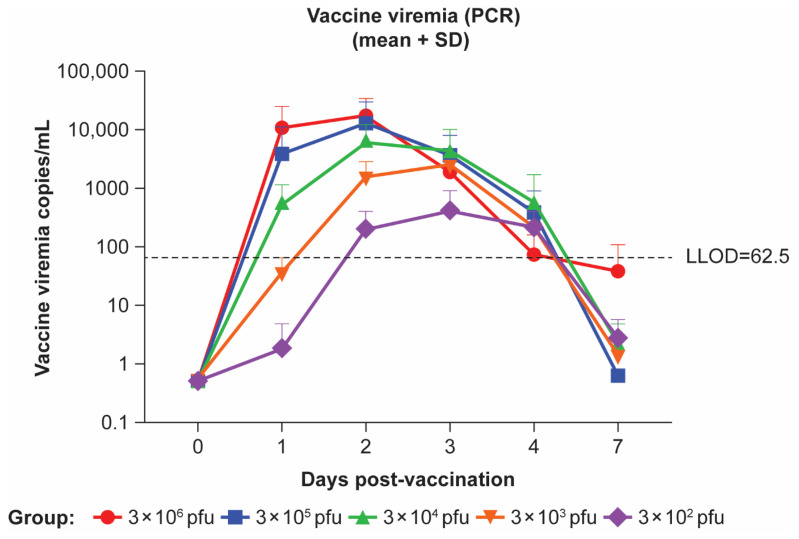
rVSVΔG-ZEBOV-GP vaccine viremia in Study 2. Five experimental groups (four or five monkeys per group) in Study 2 received 3 × 10^6^, 3 × 10^5^, 3 × 10^4^, 3 × 10^3^, or 3 × 10^2^ plaque-forming unit (pfu) intramuscular (IM) doses of rVSVΔG-ZEBOV-GP. LLOD, lower limit of detection; PCR, polymerase chain reaction; SD, standard deviation.

Vaccinated animals in Study 2 had peak rVSVΔG-ZEBOV-GP viremia on Days 2–3 post-vaccination (Figure 2). Animals vaccinated with higher doses had higher levels of vaccine viremia which were detectable earlier (Day 1) than animals vaccinated with lower doses. These levels on Day 1 post-vaccination ranged between 1189 and 30,227, 380 and 12,305, and 69 and 1219 copies/mL in the 3 × 10^6^, 3 × 10^5^, and 3 × 10^4^ pfu groups, respectively. By Day 2 post-vaccination, vaccine-associated viremia was detected in all animals with levels ranging between 1379 and 39,229, 3036 and 35,637, 2854 and 12,305, 35 and 3057, and 1 and 421 in the 3 × 10^6^, 3 × 10^5^, 3 × 10^4^, 3 × 10^3^, and 3 × 10^2^ pfu groups, respectively. Most vaccine-associated viremia was undetectable by Day 7. Statistical analysis was not conducted. Detectable levels of vaccine viremia were observed to be variable across the dose groups and among the animals within the dose groups.

### 3.4. Observations After Challenge

Forty-two days after vaccination, animals were challenged with EBOV Kikwit via IM injection (Day 0 post-challenge).

**Figure 3:**
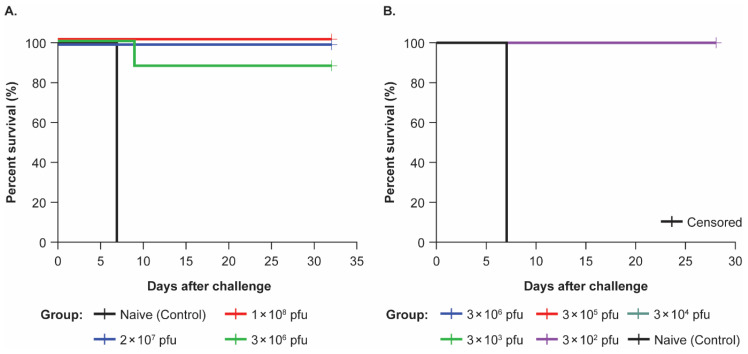
Kaplan–Meier survival curve for Ebola virus-challenged nonhuman primates. (**A**) Study 1: Includes three experimental groups that received 1 × 10^8^ (*n* = 8), 2 × 10^7^ (*n* = 7), or 3 × 10^6^ (*n* = 8) plaque-forming unit (pfu) intramuscular (IM) doses of rVSVΔG-ZEBOV-GP, and one unvaccinated naive control group (*n* = 3). One female animal in the 2 × 10^7^ pfu group was humanely euthanized during the vaccination phase and prior to the challenge dose due to gastric bloat and was not included in the time to death analysis for Ebola virus challenge. (**B**) Study 2: Includes five experimental groups that received 3 × 10^6^ (*n* = 4), 3 × 10^5^ (*n* = 4), 3 × 10^4^ (*n* = 4), 3 × 10^3^ (*n* = 5), or 3 × 10^2^ (*n* = 5) pfu IM doses of rVSVΔG-ZEBOV-GP, and one unvaccinated naive control group (*n* = 2).

In Study 1, 26 animals were challenged with an actual viral dose of 358 pfu, as verified by plaque titration of challenge inoculum. All three animals (100%) in the control group succumbed to the EBOV virus on Day 7 post-challenge and one animal in the 3 × 10^6^ vaccine group succumbed on Day 9 post-challenge (Supplementary Tables S2 and S3); all other animals (22/26) survived until the end of the in-life phase, Days 28–31 post-challenge (Figure 3A).

The four animals that succumbed in Study 1 became clinically ill (defined as having a responsiveness score of at least ‘1′) on Day 6 post-challenge, with reduced activity/responsiveness and developing rash, anorexia, and other symptoms consistent with EVD. Among the 22 animals that survived to the end of Study 1, four (two in the 1 × 10^8^ pfu group, one in the 2 × 10^7^ pfu group, and one in the 3 × 10^6^ pfu vaccine group) had a score of ‘1’ on Day 7 post-challenge, but all returned to normal health (score of ‘0’) before Day 9 post-challenge; none of the animals presented with a rash. Clinical observations included lymphadenopathy (13/22 animals; typically noted in the axillary lymph node), challenge site reaction (7/22), anorexia (7/22), and abnormal stool (6/22); however, most of these observations only lasted a day to a few days. Most vaccinated animals in the study gained weight during the challenge phase; a few animals had sporadic slight decreases in body weight noted. A majority of the animals had a slight increase in body temperature between Days 3 and 7 post-challenge. Nine of the twenty-six animals exhibited fever (≥39.5 °C), four of those animals succumbed to EBOV, while five survived (one in the 1 × 10^8^ pfu group, one in the 2 × 10^7^ pfu group, and two in the 3 × 10^6^ pfu group).

In Study 2, 24 animals were challenged with an actual viral dose of 645 pfu, as verified by plaque titration of the challenge inoculum. The two animals in the control group succumbed on Day 7 post-challenge; all other animals survived to at least 28 days post-challenge (Figure 3B). Both animals in the control group became ill on Day 6 post-challenge with both of them exhibiting a responsiveness score of up to a ‘2’ by the end of Day 6, with no urine or stool output, hemorrhage, and reduced biscuit consumption and rash from Day 5 post-challenge. One control animal was observed scoring a ‘4’ early on Day 7, meeting criteria for euthanasia first thing that morning, while the other control animal developed a score of ‘3’ by the end of Day 7, meeting euthanasia criteria at day’s end. For the animals that survived until the end of the study, five of the twenty-two animals became ill from Days 6 to 8 post-challenge (one in each of the 3 × 10^6^, (one animal had responsiveness scores of ‘1-2-1’ for 3 days) 3 × 10^5^, (one animal scored a ‘1’ for 3 days straight) and 3 × 10^3^ pfu groups (scored a ‘1’ for 1 day), and two in the 3 × 10^2^ pfu group one scored a ‘1’ for 4 days, and the other a ‘1 to 2’ for 6 days), with signs of illness including reduced activity/depressed behavior, fevers, and/or petechial rashes consistent with EVD. These animals all recovered by Day 13 post-challenge. Sixteen out of twenty-two vaccinated animals (73%) showed some level of quadriceps muscle swelling at the site of challenge, but none of these animals demonstrated responsiveness scores above ‘0’, therefore showing no signs of clinical illness. Most animals had sporadic slight decreases in body weight, with all animals in the lowest rVSVΔG-ZEBOV-GP dose group showing weight loss (<10%). Temperature elevations were observed during the post-challenge phase of the study. Six of the twenty-four animals exhibited fever; two of those in the control group succumbed to EBOV, while four survived (one in each of the 3 × 10^6^, 3 × 10^5^, 3 × 10^3^, and 3 × 10^2^ pfu groups).

### 3.5. Statistical Analysis to Predict a Correlate of Protection Threshold

In a post hoc analysis integrating the two studies, the risk of death from EBOV infection was modeled as a continuous function of immune response. The Day 28 ELISA cutoff for 50% probability of survival (C) was 978 EU/mL and the Day 28 PRNT C was 132 titer units. This analysis was repeated with C and with the maximum of C and 4× the LLOQ to account for assay variability. The bootstrap mean and 95% CI were calculated by resampling the NHP data 1000× while maintaining the original rVSVΔG-ZEBOV-GP/Placebo ratio (Table 2).

### 3.6. Antibody Responses After Challenge

To investigate vaccine-induced development of immunological memory, EBOV-specific recall response was evaluated using PsVNA in Study 2 post-challenge in a subset of eight animals (six vaccinated and two control animals), which were selected based on range of clinical responses, overall health of the animal, and other criteria (Supplementary Table S1). EBOV-specific IgG titers, as assessed by the USAMRIID non-validated ELISA, were elevated after viral challenge on Day 42 for all vaccinated animals, reaching a peak mean titer of 113,707.5 by 2 weeks post-challenge. By contrast, EBOV-specific IgG titers were not detected in the control (non-vaccinated) group by 1 week post-challenge (Supplementary Figure S2). In addition, all six vaccinated animals that had neutralizing antibody responses measured using PsVNA responded to the EBOV challenge by producing higher levels of serum neutralizing antibodies (GMT range of 10,103 to 225,863 PsVNA_50_ by Day 28 post-challenge) than those detected prior to challenge (Supplementary Figure S3). The two unvaccinated control animals did not have neutralizing antibody levels above baseline values on Day 5 post-challenge, but there was a response detected by Day 7 post-challenge (GMT range 3174 to 3485), which was most likely the neutralizing antibody response to native viral infection.

### 3.7. EBOV Viremia After Challenge

**Figure 4:**
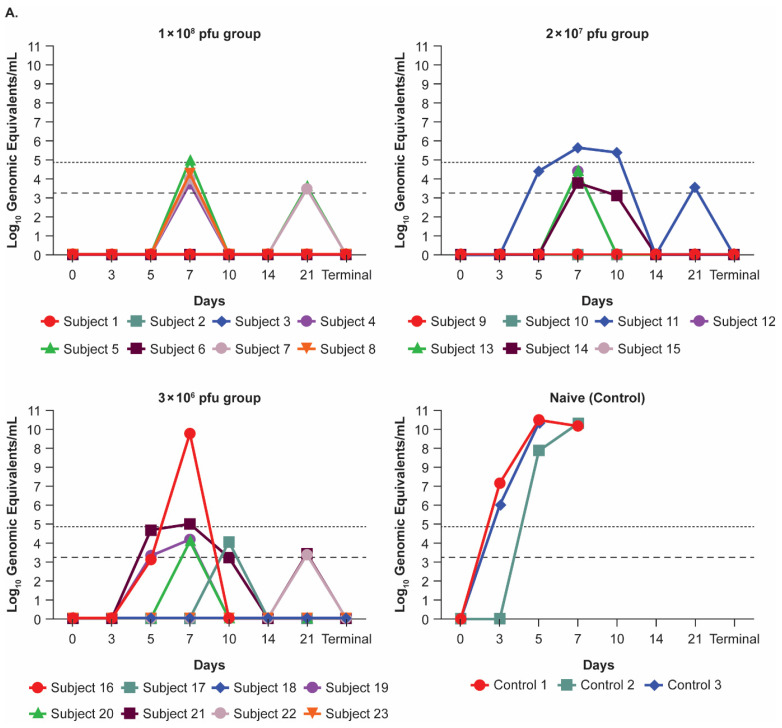

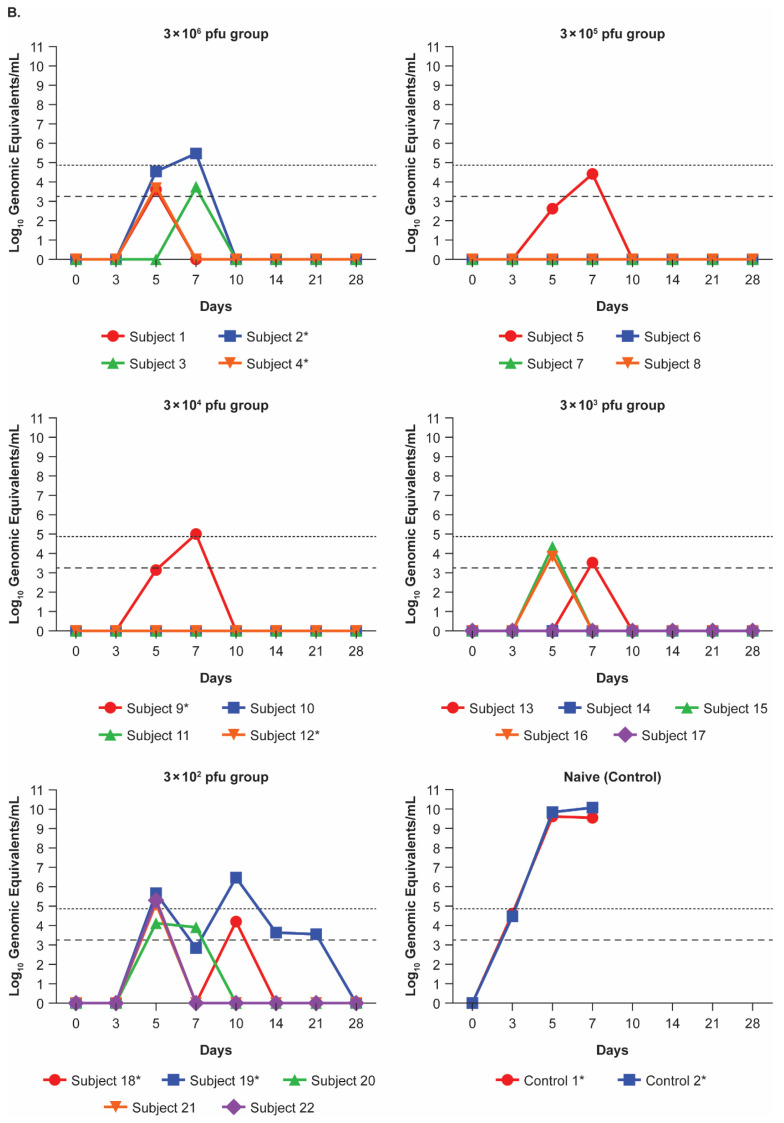
Viral load in nonhuman primates after Ebola virus challenge. (**A**) Study 1: Includes three experimental groups that received 1 × 10^8^ (*n* = 8), 2 × 10^7^ (*n* = 7), or 3 × 10^6^ (*n* = 8) plaque-forming unit (pfu) intramuscular (IM) doses of rVSVΔG-ZEBOV-GP, and one unvaccinated naive control group (*n* = 3). One female animal in the 2 × 10^7^ pfu group was humanely euthanized during the vaccination phase and prior to the challenge dose due to gastric bloat and was not included. (**B**) Study 2: Includes five experimental groups that received 3 × 10^6^ (*n* = 4), 3 × 10^5^ (*n* = 4), 3 × 10^4^ (*n* = 4), 3 × 10^3^ (*n* = 5), 3 × 10^2^ (*n* = 5) pfu IM doses of rVSVΔG-ZEBOV-GP, and one unvaccinated naive control group (*n* = 2). The upper dotted line represents the lower limit of quantitation (156.25 copies/mL) and the lower dotted line represents the lower limit of detection (62.5 copies/mL). * indicates the 8 animals that were chosen for PsVNA neutralization assay.

Some vaccinated animals in both studies had detectable EBOV viremia post-challenge but at levels significantly lower than non-vaccinated controls, and all survivors cleared the viremia. In Study 1, for the majority of vaccinated NHPs, EBOV viremia was detected but was below the LLOQ for the qRT-PCR assay (Figure 4A). By Day 5 post-challenge, all control animals had a quantifiable viral load. By Day 7 post-challenge, only four of seven vaccinated animals had a quantifiable viral load (one in the 1 × 10^8^ pfu group, one in the 2 × 10^7^ pfu group, and two in the 3 × 10^6^ pfu group). On Day 10 post-challenge, only one animal in the 2 × 10^7^ pfu group still had a quantifiable level of circulating viral genome. The vaccinated animal in the 3 × 10^6^ group that succumbed to the EBOV challenge had a quantifiable viremia on Day 7 post-challenge, peaking at levels similar to those reached by unvaccinated controls. In Study 2, only five out of twenty-two vaccinated animals had virus above the LLOQ for the assay, with viral loads much lower than levels observed in the control animals (Figure 4B). Both control animals had a quantifiable viral load by Day 5 post-challenge. By Day 5 post-challenge, three animals, all in the 3 × 10^2^ pfu group, had a quantifiable viral load. On Days 7 and 10 post-challenge, two (one in each of the 3 × 10^6^ and 3 × 10^4^ pfu groups) and one animal in the 3 × 10^2^ pfu group, respectively, had a quantifiable viral load.

### 3.8. Pathology After Challenge

Hematology and clinical chemistry in both studies, and coagulation in Study 2 (Supplementary Results), showed patterns of changes consistent with EBOV infection for those that succumbed as well as a few of the other animals, depending on the level of infection demonstrated.

In Study 1, between Days 3 and 5 post-challenge, most animals experienced a slight decrease in lymphocytes and platelet counts, particularly notable for the animals that succumbed to EBOV. A general trend of increasing lymphocytes was observed in all three vaccine groups from Day 7 to 21 post-challenge. Also, common among the vaccinated animals was a sharp reduction in circulating lymphocytes between Day 21 post-challenge and the end of the in-life phase, approximately 1 week later, with a return to normal levels. Increased platelet values were also noted in several animals in all vaccinated groups and generally occurred on Days 10 and/or 14 post-challenge.

In Study 2, lymphocyte counts decreased in the control group on Day 5 post-challenge and rebounded on Day 7 post-challenge. Lymphocyte counts were, on average, elevated in the 3 × 10^6^ and 3 × 10^5^ pfu groups across most days post-challenge relative to baseline and the lower dose groups. Lymphocyte counts were elevated across all dose groups at Days 14 and 21 post-challenge and remained elevated until Day 28 post-challenge. Platelet values were increased on average in all vaccinated groups on Day 14 post-challenge.

Typical EBOV infection results in increases in circulating aspartate aminotransferase (AST) concentrations [46]. In both studies, post-challenge AST levels were comparatively normal in the vaccine groups, with slightly elevated levels in some animals, whereas levels in the control group were substantially elevated. The maximum level of circulating AST in the vaccine groups (290 U/L) was observed in the 3 × 10^6^ pfu group on Day 10 post-challenge. In comparison, the control group had AST levels approaching 1500 U/L on Day 7 post-challenge. In Study 2, all of the animals in the vaccinated groups had comparatively normal levels of AST, whereas levels were elevated in the control animals.

Ebolavirus infections are also often associated with dramatic increases in circulating alkaline phosphatase (ALP) due to the massive amount of viral replication in the liver and resulting hepatocellular necrosis [46]. Increased ALP values were particularly prominent in the animals that succumbed to EBOV in Study 1 (one animal in the 1 × 10^8^ pfu group, and all three control animals) and in the two control animals in Study 2 (1.5-fold ALP values on Day 47, with >10-fold elevations in ALP by Day 7 post challenge (Day 49)).

### 3.9. Anatomic Pathology After Challenge

The animals that succumbed to EBOV exhibited gross, histologic, and immunohistochemical changes consistent with IM EBOV infection in NHPs. The principal tissues affected were the spleen, liver, kidney, lungs, and peripheral lymph nodes. Although the pathology lesions were similar among the non-survivors in Study 1 (*n* = 4), histologic lesions in the single vaccinated animal that died (in the 3 × 10^6^ pfu group) were slightly more widespread in distribution and severity (albeit subjectively) than lesions in the control animals. This could be due to the slightly longer survival than any of the control animals (succumbed on Day 9 vs. Day 7) or that the vaccination may have influenced the course of disease (i.e., delayed onset and/exacerbation of the disease).

For the animals that survived to termination, there were no gross necropsy findings or histologic lesions indicative of active EBOV infection, with the exception of two animals in Study 2 that had evidence of active residual infection with viral antigen detected in the inoculation site quadriceps muscle (both) and the eye (one animal). Immunohistochemistry analyses also showed immunoreactivity to Ebolavirus antigen in the muscle and eyes of these two animals in Study 2 and weak/mild immunoreactivity in the inguinal lymph nodes in two animals in Study 1 (one each in the 2 × 10^7^ pfu group and 3 × 10^6^ pfu group, with very weak extracellular staining in a few germinal centers in the spleen in the latter animal). In situ hybridization analysis of the eyes in Study 2 detected viral RNA in the eyes of three animals: in the choroid and uveal layers in both non-survivors and in the vitreous chamber adjacent to the inner limiting membrane in one survivor (in the 3 × 10^2^ group).

## 4. Discussion

At the time these studies were conducted, the 2014–2016 Western Africa Ebola outbreak was at its height. These studies were used to justify the human dose using the identical clinical-grade vaccine product and vaccination regimen and the same vaccine doses as used in human clinical trials. These data packages were submitted for regulatory agency review in support of product licensure. Testing with the same validated assays as were used to evaluate human immune responses, vaccination of NHPs with rVSVΔG-ZEBOV-GP resulted in high levels of EBOV-specific IgG measured by ELISA and neutralizing antibody titers with no dose-dependent response in either study across dose groups ranging from 1 × 10^8^ to 1 × 10^2^ pfu when evaluated after antibody responses were fully developed in all animals, after Day 7 by ELISA or after Day 14 by PRNT. Our findings demonstrated a correlation between EBOV-specific IgG titers and neutralizing antibody titers in vaccinated animals. In addition, there was more variability in magnitude of responses among animals at lower vaccine doses, also observed in human Phase 1 dose ranging studies [13,14,15,16,17,18,19]. The lack of a dose response at later timepoints in our studies was likely due to the replicating nature of the live attenuated vaccine, with all except one vaccinated animal fully protected against death by the dose received. Antibodies appear to have a critical role in rVSV-mediated protection against EBOV as shown in the study by Marzi et al., in which depletion of CD8+ T cells had no impact on survival but the depletion of CD4+ T cells during vaccination with rVSVΔG-ZEBOV-GP in macaques resulted in no IgG response and no protection from subsequent EBOV challenge [36]. Robust immunological responses have been demonstrated in clinical trials in healthy adults, although with dose-dependent responses observed in these clinical trials using similar vaccine doses [14,17,18,19,47]. A study using an rVSV-based vaccine expressing the EBOV-Makona GP in the NHP model also titrated the vaccine and observed complete protection from challenge with 1000 LD50s Makona strain of Ebola virus at vaccine doses ranging from 1 × 10^1^ to 1 × 10^7^ pfu, with no dose-dependent effects on IgG titers or neutralizing responses observed in surviving animals (although lower doses of 1 × 10^0^ and 1 × 10^−1^ did not provide complete protection) [48]. Interestingly, an NHP study with rVSVΔG-ZEBOV-GP observed higher soluble glycoprotein-binding antibody levels and higher GP-ELISA titers in animals that survived EBOV challenge [49]. The results of our NHP study indicate that although there may be differences in the kinetics and/or consistency of the immune responses associated with lower doses of vaccine, the replicating vaccine is able to protect NHPs across a very wide dose range. In humans, however, dose may have a higher impact, as differences in immune responses are detected even at Day 28 post-vaccination in some studies [17]. The difference in immune responses between humans and macaques warrants further investigation.

Across these two NHP studies, 44/45 EBOV challenged animals survived (97.8% survival) after immunization with the subsequently licensed clinical-grade rVSVΔG-ZEBOV-GP vaccine across a wide range of dose groups. In Study 1, all animals in the 1 × 10^8^ and 2 × 10^7^, and seven out of eight animals in the 3 × 10^6^ pfu vaccine dose groups, survived challenge to the end of the study. The one non-surviving vaccinated animal succumbed on Day 9 post-challenge, although this animal had a robust IgG titer prior to challenge, similar to that of vaccinated survivors. In addition, one animal in the 2 × 10^7^ pfu group was humanely euthanized during the vaccination phase due to gastric bloat (which occurs sporadically in captive NHPs in preclinical studies [45]). Based on Study 1 results, the dose deescalation design of Study 2 was implemented to evaluate the possibility of determining a breakthrough vaccine dose in NHP, which might yield a suboptimally vaccinated animal group allowing the study of correlates of immune protection. However, in Study 2, all of the vaccinated animals survived, and even the lowest dose of vaccine, 3 × 10^2^ pfu, was fully protective against death (but not disease) in this challenge model. Previous NHP studies evaluating the research-grade rVSVΔG-ZEBOV-GP vaccine at a single dose level similar to that used in Study 1, approximately 1 × 10^7^ pfu, showed similar efficacy, with 100% effectiveness when delivered >14 days prior to challenge with EBOV [32,33,34,35]. The 2 × 10^7^ pfu dose used in Study 1 was selected for Phase 2/3 trials based in part on the NHP data in Study 1 [48,50] as well as the Phase 1 trials conducted in parallel [17]. Human clinical efficacy of the vaccine when administered at a dose of 2 × 10^7^ pfu was demonstrated in the Phase 3 ring vaccination trial (Ebola Ça Suffit) conducted in Guinea in early 2015 [22]. Integrated analysis of both NHP studies, including controls, demonstrated that higher ELISA titers on Day 35 post-vaccination were associated with a lower odds ratio for death upon challenge on Day 42. With robust antibody responses at all doses and so few non-survivors in this study, a threshold level of antibody response that predicts survival or time to death could not be determined from the initial analysis of these data where survival was the outcome and dose or titer response was the predictor using a Cox proportional hazards model and logistic regression. Subsequent post hoc analysis using logistic regression to model the risk of death from EBOV infection in NHP as a continuous function of immune response indicated a Day 28 cutoff for 50% probability of survival for ELISA was 978 EU/mL and for PRNT was 132 titer units. Antibody titers in NHP are notably higher than in humans receiving the same or similar dose, yet efficacy is similarly high in both species, suggesting that antibody titers below these thresholds are likely protective in NHP. Application of the threshold previously reported for human ELISA responses that best distinguishes between vaccine and placebo recipients, at least 200 EU/mL and at least 2-fold increase over baseline [51], is strongly associated with protection from EBOV challenge in these NHP studies. This is consistent with a recent post hoc analysis of data from the rVSVΔG-ZEBOV-GP Phase 2/3 clinical trials suggesting a dichotomous correlate of protection for humans using this seroresponse definition [43]. Future studies and analyses exploring other immune response measures (such as Fc effector function, antibody affinity, and epitope specificity) may provide additional insights into a potential immune correlate threshold of protection.

Few, if any, signs of EBOV disease were observed in any of the vaccinated animals post-challenge, but a trend toward more viremia and signs of disease were noted at lower doses despite overall survival; post-challenge injection-site reactions (quadriceps muscle swelling—likely immune response contained EBOV at site of challenge and not disseminated EVD) occurred in seven of twenty-two animals in Study 1 and sixteen of twenty-two animals in Study 2. In Study 2, all of the vaccinated animals had similar high titer anti-EBOV GP antibody responses post-challenge, and the six vaccinated animals tested for neutralizing antibodies all had higher levels of antibodies post-challenge. This was consistent with robust anamnestic responses following challenge and demonstrated that the vaccine did not induce sterilizing immunity but was able to contain the virus and prevent disease and death.

For the majority of vaccinated animals across both studies, EBOV viral RNA after challenge was below the LLOQ of the qRT-PCR assay. The vaccinated animal that succumbed to EBOV challenge in Study 1 had a quantifiable viremia on Day 7 post-challenge, peaking at levels similar to those of unvaccinated controls on Day 5 post-challenge. Only eight out of forty-three vaccinated animals who survived had virus above the lower limit of quantitation, all at much lower levels than observed in the control animals. Many of the remaining animals had a transient, low level of detectable EBOV RNA, below the lower limit of quantitation for the assay. There was no clear vaccine–dose relationship to EBOV replication levels.

Pathology parameters showed patterns of change consistent with EBOV infection in the non-survivors and in a few of the vaccinated animals with higher levels of infection. Among the non-survivors that succumbed to EBOV, the observed gross, histologic, and immunohistochemical changes were also consistent with IM EBOV infection in NHPs. A possible explanation for the slightly more widespread and severe histologic lesions in the one vaccinated non-survivor may be that this vaccinated animal survived slightly longer than any of the control animals or that the vaccination may have influenced the course of the disease (e.g., delayed onset). Two vaccinated animals in Study 2 had evidence of viral antigen in the eye or the muscle at the virus inoculation site, but plasma viremias were resolved in these survivors. Long-term persistence of EBOV after infection has been documented in immune-privileged organs of human survivors [46,52,53,54,55].

In summary, vaccination with rVSVΔG-ZEBOV-GP induced high levels of EBOV-specific IgG and neutralizing antibody titers with no dose response observed. Notably, after Day 14 post-vaccination, the identical validated assays used in clinical trials of the now licensed vaccine were employed in these evaluations. Vaccination with rVSVΔG-ZEBOV-GP conferred 97.8% protection against lethal EBOV challenge, with 44/45 challenged animals surviving across all dose groups ranging from as low as 3 × 10^2^ up to 1 × 10^8^ pfu across both studies. All control animals succumbed on Day 7, while only one vaccinated animal succumbed, on Day 9 post-challenge. Although a specific antibody threshold predicting survival or time to death could not be identified from the initial analysis, post hoc analysis suggested that lower antibody titers may still be protective (an ELISA titer of at least 200 EU/mL or a 2-fold increase over baseline is strongly associated with protection). These findings align with clinical trial data indicating correlates of protection in humans and reinforce the robust immune response and high level of protection conferred by rVSVΔG-ZEBOV-GP vaccination.

## Figures and Tables

**Table 1:** Overview of the two immunogenicity and efficacy studies of rVSVΔG-ZEBOV-GP in NHPs.

Study No.	Type of Study	Species	No. per Group	Dose Regimen of Vaccination	Challenge
Study 1	Immunogenicity, efficacy and dose selection study	Cynomolgus macaques	8 per treatment group; 3 control animals * (27 total animals)	One dose of 1 mL IM at 1 × 10^8^, 2 × 10^7^, or 3 × 10^6^ pfu	IM challenge 42 days after vaccination (1 mL, target dose 1000 pfu)
Study 2	Dose de-escalation immunogenicity, efficacy	Cynomolgus macaques	4 to 5 per treatment group; 2 control animals * (24 total animals)	One dose of 1 mL IM at 3 × 10^6^, 3 × 10^5^, 3 × 10^4^, 3 × 10^3^, or 3 × 10^2^ pfu	IM challenge 42 days after vaccination (0.5 mL, target dose 1000 pfu)

* Control groups in both studies received diluent/saline only. IM, intramuscular; pfu, plaque-forming units; rVSVΔG-ZEBOV-GP, recombinant vesicular stomatitis virus-Zaire Ebolavirus envelope glycoprotein vaccine. Vaccine and challenge virus.

**Table 2:** Predicted cutoff for 50% probability of survival.

Day 28 Endpoint	All Data	Max [Cutoff, 4× LLOQ] (95% CI)	Cutoff (95% CI)
ELISA (EU/mL)	978	1199 (355–4864)	1199 (355–4864)
PRNT (titer units)	132	165 (80–470)	162 (60–470)

Cutoff = cutoff for 50% probability of survival; ELISA LLOQ = 13.62; PRNT LLOQ = 20. CI, confidence interval; ELISA, enzyme-linked immunosorbent assay; EU, ELISA units; LLOQ, lower limit of quantitation; PRNT, plaque reduction neutralization test.

## Data Availability

The data sharing policy, including restrictions, of Merck Sharp & Dohme LLC, a subsidiary of Merck & Co., Inc., Rahway, NJ, USA (MSD), is available at https://trialstransparency.msdclinicaltrials.com/policies-perspectives.aspx (accessed on 15 January 2025). Requests for access to the clinical study data can be submitted via email to the Data Access mailbox (mail to: dataaccess@msd.com).

## Supplementary Materials

The following supporting information can be downloaded at: https://www.mdpi.com/article/10.3390/v17030341/s1, Table S1. Selection criteria for pseudovirion neutralization assay (PsVNA). Table S2. Antibody titers post-vaccination from the one vaccinated NHP who succumbed on Day 9 post-challenge. Table S3. Viral load post-challenge from the one vaccinated NHP who succumbed on Day 9 post-challenge. Figure S1. Antibody responses in nonhuman primates after single intramuscular administration of various doses of rVSVΔG-ZEBOV-GP determined using non-validated assays. Figure S2. Ebolavirus-glycoprotein (EBOV-GP)–specific immunoglobulin G antibody titers in nonhuman primates after single intramuscular administration of various doses of rVSVΔG-ZEBOV-GP in Study 2. Figure S3. Neutralizing antibody activity before and after challenge as measured by PsVNA_50_ in Study 2.

## References

[1] World Health Organization (WHO) Ebola Virus Disease. https://www.who.int/en/news-room/fact-sheets/detail/ebola-virus-disease.

[2] Den Boon S., Marston B.J., Nyenswah T.G., Jambai A., Barry M., Keita S., Durski K., Senesie S.S., Perkins D., Shah A. (2019). Ebola Virus Infection Associated with Transmission from Survivors. Emerg. Infect. Dis..

[3] Gupta M., Mahanty S., Greer P., Towner J.S., Shieh W.J., Zaki S.R., Ahmed R., Rollin P.E. (2004). Persistent infection with ebola virus under conditions of partial immunity. J. Virol..

[4] Thorson A.E., Deen G.F., Bernstein K.T., Liu W.J., Yamba F., Habib N., Sesay F.R., Gaillard P., Massaquoi T.A., McDonald S.L.R. (2021). Persistence of Ebola virus in semen among Ebola virus disease survivors in Sierra Leone: A cohort study of frequency, duration, and risk factors. PLoS Med..

[5] Centers for Disease Control and Prevention (CDC) Outbreak History. https://www.cdc.gov/ebola/outbreaks/index.html.

[6] Centers for Disease Control and Prevention (CDC) Ebola Disease Basics. https://www.cdc.gov/ebola/about/index.html.

[7] Lefebvre A., Fiet C., Belpois-Duchamp C., Tiv M., Astruc K., Aho Glele L.S. (2014). Case fatality rates of Ebola virus diseases: A meta-analysis of World Health Organization data. Med. Mal. Infect..

[8] U.S. Food and Drug Administration (2020). FDA Approves Treatment for Ebola Virus [Press Release]. https://www.fda.gov/drugs/news-events-human-drugs/fda-approves-treatment-ebola-virus.

[9] U.S. Food and Drug Administration (2020). FDA Approves First Treatment for Ebola Virus [Press Release]. https://www.fda.gov/news-events/press-announcements/fda-approves-first-treatment-ebola-virus.

[10] Mulangu S., Dodd L.E., Davey R.T., Tshiani Mbaya O., Proschan M., Mukadi D., Lusakibanza Manzo M., Nzolo D., Tshomba Oloma A., Ibanda A. (2019). A Randomized, Controlled Trial of Ebola Virus Disease Therapeutics. N. Engl. J. Med..

[11] Monath T.P., Fast P.E., Modjarrad K., Clarke D.K., Martin B.K., Fusco J., Nichols R., Heppner D.G., Simon J.K., Dubey S. (2019). rVSVΔG-ZEBOV-GP (also designated V920) recombinant vesicular stomatitis virus pseudotyped with Ebola Zaire Glycoprotein: Standardized template with key considerations for a risk/benefit assessment. Vaccine X.

[12] Coller B.G., Lapps W., Yunus M., Bruno S., Eichberg M.J., Lee A.W., Liu K., Drury R., Millogo J., Macareo L.R. (2022). Lessons Learned from the Development and Roll-Out of the rVSVΔG-ZEBOV-GP Zaire ebolavirus Vaccine to Inform Marburg Virus and Sudan ebolavirus Vaccines. Vaccines.

[13] Agnandji S.T., Fernandes J.F., Bache E.B., Obiang Mba R.M., Brosnahan J.S., Kabwende L., Pitzinger P., Staarink P., Massinga-Loembe M., Krähling V. (2017). Safety and immunogenicity of rVSVΔG-ZEBOV-GP Ebola vaccine in adults and children in Lambaréné, Gabon: A phase I randomised trial. PLoS Med..

[14] Agnandji S.T., Huttner A., Zinser M.E., Njuguna P., Dahlke C., Fernandes J.F., Yerly S., Dayer J.A., Kraehling V., Kasonta R. (2016). Phase 1 Trials of rVSV Ebola Vaccine in Africa and Europe. N. Engl. J. Med..

[15] Dahlke C., Kasonta R., Lunemann S., Krähling V., Zinser M.E., Biedenkopf N., Fehling S.K., Ly M.L., Rechtien A., Stubbe H.C. (2017). Dose-dependent T-cell Dynamics and Cytokine Cascade Following rVSV-ZEBOV Immunization. EBioMedicine.

[16] ElSherif M.S., Brown C., MacKinnon-Cameron D., Li L., Racine T., Alimonti J., Rudge T.L., Sabourin C., Silvera P., Hooper J.W. (2017). Canadian Immunization Research Network, Assessing the safety and immunogenicity of recombinant vesicular stomatitis virus Ebola vaccine in healthy adults: A randomized clinical trial. CMAJ.

[17] Heppner D.G., Kemp T.L., Martin B.K., Ramsey W.J., Nichols R., Dasen E.J., Link C.J., Das R., Xu Z.J., Sheldon E.A. (2017). Safety and immunogenicity of the rVSVΔG-ZEBOV-GP Ebola virus vaccine candidate in healthy adults: A phase 1b randomised, multicentre, double-blind, placebo-controlled, dose-response study. Lancet Infect. Dis..

[18] Huttner A., Dayer J.A., Yerly S., Combescure C., Auderset F., Desmeules J., Eickmann M., Finckh A., Goncalves A.R., Hooper J.W. (2015). The effect of dose on the safety and immunogenicity of the VSV Ebola candidate vaccine: A randomised double-blind, placebo-controlled phase 1/2 trial. Lancet Infect. Dis..

[19] Regules J.A., Beigel J.H., Paolino K.M., Voell J., Castellano A.R., Hu Z., Munoz P., Moon J., Ruck R., Bennet J. (2017). rVSVΔG-ZEBOV-GP Study Group, A Recombinant Vesicular Stomatitis Virus Ebola Vaccine. N. Engl. J. Med..

[20] Halperin S.A., Arribas J.R., Rupp R., Andrews C.P., Chu L., Das R., Simon J.K., Onorato M.T., Liu K., Martin J. (2017). Six-Month Safety Data of Recombinant Vesicular Stomatitis Virus-Zaire Ebola Virus Envelope Glycoprotein Vaccine in a Phase 3 Double-Blind, Placebo-Controlled Randomized Study in Healthy Adults. J. Infect. Dis..

[21] Halperin S.A., Das R., Onorato M.T., Liu K., Martin J., Grant-Klein R.J., Nichols R., Coller B.A., Helmond F.A., Simon J.K. (2019). V920-012 Study Team, Immunogenicity, Lot Consistency, and Extended Safety of rVSVΔG-ZEBOV-GP Vaccine: A Phase 3 Randomized, Double-Blind, Placebo-Controlled Study in Healthy Adults. J. Infect. Dis..

[22] Henao-Restrepo A.M., Camacho A., Longini I.M., Watson C.H., Edmunds W.J., Egger M., Carroll M.W., Dean N.E., Diatta I., Doumbia M. (2017). Efficacy and effectiveness of an rVSV-vectored vaccine in preventing Ebola virus disease: Final results from the Guinea ring vaccination, open-label, cluster-randomised trial (Ebola Ca Suffit!). Lancet.

[23] Kennedy S.B., Bolay F., Kieh M., Grandits G., Badio M., Ballou R., Eckes R., Feinberg M., Follmann D., Grund B. (2017). Phase 2 Placebo-Controlled Trial of Two Vaccines to Prevent Ebola in Liberia. N. Engl. J. Med..

[24] Samai M., Seward J.F., Goldstein S.T., Mahon B.E., Lisk D.R., Widdowson M.A., Jalloh M.I., Schrag S.J., Idriss A., Carter R.J. (2018). The Sierra Leone Trial to Introduce a Vaccine Against Ebola: An Evaluation of rVSVΔG-ZEBOV-GP Vaccine Tolerability and Safety During the West Africa Ebola Outbreak. J. Infect. Dis..

[25] Huttner A., Agnandji S.T., Engler O., Hooper J.W., Kwilas S., Ricks K., Clements T.L., Jonsdottir H.R., Nakka S.S., Rothenberger S. (2023). Antibody responses to recombinant vesicular stomatitis virus-Zaire Ebolavirus vaccination for Ebola virus disease across doses and continents: 5-year durability. Clin. Microbiol. Infect..

[26] Kieh M., Richert L., Beavogui A.H., Grund B., Leigh B., D’Ortenzio E., Doumbia S., Lhomme E., Sow S., Prevac Study Team (2022). Randomized Trial of Vaccines for Zaire Ebola Virus Disease. N. Engl. J. Med..

[27] European Medicines Agency Ervebo: EPAR—Product Information. https://www.ema.europa.eu/en/medicines/human/EPAR/ervebo.

[28] U.S. Food and Drug Administration First FDA-Approved Vaccine for the Prevention of Ebola Virus Disease, Marking a Critical Milestone in Public Health Preparedness and Response. https://www.fda.gov/news-events/press-announcements/first-fda-approved-vaccine-prevention-ebola-virus-disease-marking-critical-milestone-public-health.

[29] (2023). ERVEBO^®^ Prescribing Information.

[30] Kallay R., Doshi R.H., Muhoza P., Choi M.J., Legand A., Aberle-Grasse E., Bagayoko A., Hyde T.B., Formenty P., Costa A. (2024). Use of Ebola Vaccines—Worldwide, 2021–2023. MMWR Morb. Mortal. Wkly. Rep..

[31] European Medicines Agency New Vaccine for Prevention of Ebola Virus Disease Recommended for Approval in the European Union. https://www.ema.europa.eu/en/news/new-vaccine-prevention-ebola-virus-disease-recommended-approval-european-union.

[32] Geisbert T.W., Daddario-DiCaprio K.M., Lewis M.G., Geisbert J.B., Grolla A., Leung A., Paragas J., Matthias L., Smith M.A., Jones S.M. (2008). Vesicular stomatitis virus-based ebola vaccine is well-tolerated and protects immunocompromised nonhuman primates. PLoS Pathog..

[33] Geisbert T.W., Geisbert J.B., Leung A., Daddario-DiCaprio K.M., Hensley L.E., Grolla A., Feldmann H. (2009). Single-injection vaccine protects nonhuman primates against infection with marburg virus and three species of ebola virus. J. Virol..

[34] Jones S.M., Feldmann H., Stroher U., Geisbert J.B., Fernando L., Grolla A., Klenk H.D., Sullivan N.J., Volchkov V.E., Fritz E.A. (2005). Live attenuated recombinant vaccine protects nonhuman primates against Ebola and Marburg viruses. Nat. Med..

[35] Qiu X., Fernando L., Alimonti J.B., Melito P.L., Feldmann F., Dick D., Stroher U., Feldmann H., Jones S.M. (2009). Mucosal immunization of cynomolgus macaques with the VSVΔG/ZEBOVGP vaccine stimulates strong ebola GP-specific immune responses. PLoS ONE.

[36] Marzi A., Engelmann F., Feldmann F., Haberthur K., Shupert W.L., Brining D., Scott D.P., Geisbert T.W., Kawaoka Y., Katze M.G. (2013). Antibodies are necessary for rVSV/ZEBOV-GP-mediated protection against lethal Ebola virus challenge in nonhuman primates. Proc. Natl. Acad. Sci. USA.

[37] Kugelman J.R., Rossi C.A., Wiley M.R., Ladner J.T., Nagle E.R., Pfeffer B.P., Garcia K., Prieto K., Wada J., Kuhn J.H. (2016). Informing the Historical Record of Experimental Nonhuman Primate Infections with Ebola Virus: Genomic Characterization of USAMRIID Ebola Virus/H.sapiens-tc/COD/1995/Kikwit-9510621 Challenge Stock “R4368” and Its Replacement “R4415”. PLoS ONE.

[38] Trefry J.C., Wollen S.E., Nasar F., Shamblin J.D., Kern S.J., Bearss J.J., Jefferson M.A., Chance T.B., Kugelman J.R., Ladner J.T. (2015). Ebola Virus Infections in Nonhuman Primates Are Temporally Influenced by Glycoprotein Poly-U Editing Site Populations in the Exposure Material. Viruses.

[39] Geisbert T.W., Strong J.E., Feldmann H. (2015). Considerations in the Use of Nonhuman Primate Models of Ebola Virus and Marburg Virus Infection. J. Infect. Dis..

[40] Shurtleff A.C., Bloomfield H.A., Mort S., Orr S.A., Audet B., Whitaker T., Richards M.J., Bavari S. (2016). Validation of the Filovirus Plaque Assay for Use in Preclinical Studies. Viruses.

[41] Rudge T.L., Sankovich K.A., Niemuth N.A., Anderson M.S., Badorrek C.S., Skomrock N.D., Cirimotich C.M., Sabourin C.L. (2019). Development, qualification, and validation of the Filovirus Animal Nonclinical Group anti-Ebola virus glycoprotein immunoglobulin G enzyme-linked immunosorbent assay for human serum samples. PLoS ONE.

[42] Niemuth N.A., Rudge T.L., Sankovich K.A., Anderson M.S., Skomrock N.D., Badorrek C.S., Sabourin C.L. (2020). Method feasibility for cross-species testing, qualification, and validation of the Filovirus Animal Nonclinical Group anti-Ebola virus glycoprotein immunoglobulin G enzyme-linked immunosorbent assay for non-human primate serum samples. PLoS ONE.

[43] Grais R.F., Kennedy S.B., Mahon B.E., Dubey S.A., Grant-Klein R.J., Liu K., Hartzel J., Coller B.A., Welebob C., Hanson M.E. (2021). Estimation of the correlates of protection of the rVSVΔG-ZEBOV-GP Zaire ebolavirus vaccine: A post-hoc analysis of data from phase 2/3 clinical trials. Lancet Microbe.

[44] Warren T., Zumbrun E., Weidner J.M., Gomba L., Rossi F., Bannister R., Tarrant J., Reed M., Lee E., Raymond J.L. (2020). Characterization of Ebola Virus Disease (EVD) in Rhesus Monkeys for Development of EVD Therapeutics. Viruses.

[45] Parrott T. Miscellaneous Conditions of Nonhuman Primates. https://www.msdvetmanual.com/exotic-and-laboratory-animals/nonhuman-primates/miscellaneous-conditions-of-nonhuman-primates.

[46] Schieffelin J.S., Shaffer J.G., Goba A., Gbakie M., Gire S.K., Colubri A., Sealfon R.S., Kanneh L., Moigboi A., Momoh M. (2014). Clinical illness and outcomes in patients with Ebola in Sierra Leone. N. Engl. J. Med..

[47] Huttner A., Agnandji S.T., Combescure C., Fernandes J.F., Bache E.B., Kabwende L., Ndungu F.M., Brosnahan J., Monath T.P., Lemaitre B. (2018). Determinants of antibody persistence across doses and continents after single-dose rVSV-ZEBOV vaccination for Ebola virus disease: An observational cohort study. Lancet Infect. Dis..

[48] Marzi A., Reynolds P., Mercado-Hernandez R., Callison J., Feldmann F., Rosenke R., Thomas T., Scott D.P., Hanley P.W., Haddock E. (2019). Single low-dose VSV-EBOV vaccination protects cynomolgus macaques from lethal Ebola challenge. EBioMedicine.

[49] Gunn B.M., McNamara R.P., Wood L., Taylor S., Devadhasan A., Guo W., Das J., Nilsson A., Shurtleff A., Dubey S. (2023). Antibodies against the Ebola virus soluble glycoprotein are associated with long-term vaccine-mediated protection of non-human primates. Cell Rep..

[50] Marzi A., Robertson S.J., Haddock E., Feldmann F., Hanley P.W., Scott D.P., Strong J.E., Kobinger G., Best S.M., Feldmann H. (2015). VSV-EBOV rapidly protects macaques against infection with the 2014/15 Ebola virus outbreak strain. Science.

[51] Antonello J., Grant-Klein R.J., Nichols R., Kennedy S.B., Dubey S., Simon J.K. (2020). Serostatus cutoff levels and fold increase to define seroresponse to recombinant vesicular stomatitis virus–Zaire Ebola virus envelope glycoprotein vaccine: An evidence-based analysis. Vaccine.

[52] Varkey J.B., Shantha J.G., Crozier I., Kraft C.S., Lyon G.M., Mehta A.K., Kumar G., Smith J.R., Kainulainen M.H., Whitmer S. (2015). Persistence of Ebola Virus in Ocular Fluid during Convalescence. N. Engl. J. Med..

[53] Deen G.F., Broutet N., Xu W., Knust B., Sesay F.R., McDonald S.L.R., Ervin E., Marrinan J.E., Gaillard P., Habib N. (2017). Ebola RNA Persistence in Semen of Ebola Virus Disease Survivors–Final Report. N. Engl. J. Med..

[54] Schindell B.G., Webb A.L., Kindrachuk J. (2018). Persistence and Sexual Transmission of Filoviruses. Viruses.

[55] Jacob S.T., Crozier I., Fischer W.A., Hewlett A., Kraft C.S., Vega M.A., Soka M.J., Wahl V., Griffiths A., Bollinger L. (2020). Ebola virus disease. Nat. Rev. Dis. Primers.

