# Supplementary material for: rVSVΔG-ZEBOV-GP Vaccine Is Highly Immunogenic and Efficacious Across a Wide Dose Range in a Nonhuman Primate EBOV Challenge Model
Source: Viruses. 2025 Feb 28;17(3):341. doi: 10.3390/v17030341 (PMC11945660; doi:10.3390/v17030341)
Supplement: Supplementary file 1 [file viruses-17-00341-s001.zip › viruses-3457162-supplementary.pdf]

rVSVΔG-ZEBOV-GP Vaccine is Highly Immunogenic and Efficacious Across a Wide Dose Range in a Nonhuman Primate EBOV Challenge Model. Shurtleff et al.

SUPPLEMENTARY MATERIALS

SUPPLEMENTARY METHODS

Immune response assays

Non-validated ELISA

For collection of interim data for informational purposes, serum samples were tested at USAMRIID for EBOV-GP–specific antibodies using a non-validated ELISA. Briefly, IAW USAMRIID SOP AP-03-36 Immunolon 2HB, 96-well microtiter plates (Thermo Fisher) were pre-coated with 0.5 µg/mL recombinant Zaire Kikwit GP (Advanced BioScience Laboratories, Inc.) in 1× phosphate buffered saline (PBS). Coated plates were stored at 2–8°C for 14 hours to 5 days.

Serum samples were initially diluted in titer tubes. They were further two-fold serially diluted in titer tubes, as appropriate, to allow for the endpoint titer of the sample to be determined. The starting dilution put on the plate was 1:50 for most samples. For samples whose endpoint was not determined with a 1:50 plate starting dilution, or those whose earlier time point did not reach endpoint with a 1:50 plate starting dilution, the initial starting dilution tested on the plate was increased to 1:400. Generally, ten dilutions per sample were tested on the ELISA plates. For higher titer samples the number of dilutions tested was increased, as needed, to ensure detection of endpoint titers.

The reference standard was an NHP reference sample called RMR 1404, BMIZAIRE007, with a known concentration of 1,104.58 EU/mL. The reference standard curve was prepared by making a 1:100 starting dilution of the reference standard, followed by 10 subsequent two-fold serial dilutions, for a total of 11 points in the standard curve. The quality control (QC) samples, RMR 1402, BMIZAIRE004 (high QC) and RMR 1403 BMIZAIRE005 (low QC),were prepared with a 1:50 starting dilution, and five subsequent two-fold serial dilutions, for a total of six test dilutions. The negative control sample was

diluted to 1:50. All dilutions were made in a solution of 1× PBS, 5% skim milk, and 0.1% Tween 20 (Sigma, Lab Scientific, and Sigma, respectively).

The pre-coated plates were washed three times, using 1× PBS with 0.1% Tween 20, prior to use in the assay. Standards, controls, and samples were added to the plates, sealed, and the samples allowed to react at 37±2°C for 1 hour ± 5 minutes. EBOV-GP–specific antibodies were detected using anti-NHP immunoglobulin G (IgG) secondary antibody, conjugated to horseradish peroxidase (HRP) (43R-IG020HRP, Fitzgerald), at a 1:26,000 dilution. The plates were washed prior to the addition of the secondary conjugate. The plates were sealed, and the secondary conjugate was allowed to bind at 37±2°C for 1 hour ± 5 minutes.

Antibody titers were determined based on the colorimetric product formed from HRP conjugate reacting with added peroxidase substrate 3,3',5,5-tetramethylbenzidine (TMB). The reaction was stopped 8 to 12 minutes thereafter using the manufacturer's associated stop solution (both Thermo Scientific). The plate was then read at an absorbance wavelength of 450 nm and a reference wavelength of 630 nm on a BioTek ELx808 Plate Reader (in biosafety level [BSL]-2) or a SpectraMax M5 (in BSL-4, unvalidated). For data generated on the SpectraMax M5 in BSL-4 (SoftMax Pro Software, v5.4.1), the resulting final OD (OD_{450}-OD_{630}) was determined in Microsoft Excel (Excel 2010) and then imported into the BioTek ELx808 Plate Reader for subsequent analysis. This was done to maintain continuity in the algorithms used for data generation.

The relative amount of EBOV-GP–specific antibody in a given serum sample is directly proportional to the reference wavelength-corrected absorbance value, and is calculated by interpolation from the reference standard curve using a 4-parameter logistic model generated by the GEN5 v2.07 software. The assay results reported, per NHP, are the endpoint titers and the mean concentration of back-calculated anti–EBOV-GP–specific IgG antibody levels in serum as ELISA units/mL (EU/mL).

Pseudovirion neutralization assay (PsVNA)

Pseudovirion neutralization assay was performed as previously described [1]. Pseudovirions (PsV) were produced in 293T cells transfected with plasmids encoding the EBOV GP (pWRG7077-Ebola-95) and infected with the VSV-luciferase ΔG virus. Ebola PsVs (4000 forming units) were combined with serial-diluted, heat inactivated (56°C, 30 minutes) serum overnight in the presence of 5% human complement. The PsV + serum mixture was then added to Vero-76 cell monolayers in black, clear bottom 96-well microtiter plates. The plates were incubated for 18–24 hours, media removed, lysis luciferase reagent (Promega, Madison, WI) added, and flash luminescence data acquired using a luminometer. If serum samples contained antibodies that prevent the PsV from attaching to and/or entering cells, then the reporter activity was neutralized. Neutralization titers were interpolated from 4-parameter curves using GraphPad Prism. The reciprocal of the interpolated dilution that resulted in a 50% decrease in luciferase activity was the $PsVNA_{50}$ titer.

SUPPLEMENTARY RESULTS

Pseudovirion neutralization assay and ELISA

In Study 1, the neutralizing antibody titers in all vaccinated NHPs as measured by PsVNA, regardless of dose, were relatively high (i.e., $PsVNA_{50}$ >1000) and appeared to peak on Day 28 (Supplementary Figure S1). Although group PsVNA geometric mean titers (GMTs) were not statistically compared, there was a less than 3-fold difference between GMTs for the three different vaccine cohorts. The $PsVNA_{50}$ titer was relatively low in the non-survivor in the 3×10^6 group; however, other animals that had lower titers pre-challenge survived in this study. In Study 2, a subset of eight animals that displayed a post-challenge range of clinical signs of EBOV disease (from little or none to several serious signs) were tested for neutralizing antibody activity, six of which were vaccinated. Of these, all vaccinated animals had detectable neutralizing antibodies before challenge (GMT range 3108–10,578 $PsVNA_{50}$ on Day 35) (Supplementary Figure S2). Sera from the vaccinated NHPs in Study 2

collected pre- and post-challenge were measured by ELISA (Supplementary Figure S3). Antibody in the vaccinated animals rose and peaked within 2 weeks (Day 56) after challenge, and then declined.)

Coagulation parameters after challenge

EBOV disease-related consumptive coagulopathy was observed in the samples from the two non-vaccinated control animals. Observations such as prolonged prothrombin time (PT), prolonged activated partial thromboplastin time (APTT), and prolonged thrombin time (TT) were seen in these animals compared to the vaccinated animals. From the hematology results, the control animals had reduced numbers of platelets (thrombocytopenia), which impacted clotting factor levels and clotting times.

For the vaccinated groups, PT, APTT, and TT were generally not prolonged above baseline times, and these groups' times for these factors were indistinguishable across the dose-levels of vaccine given. One animal in the 3×10^2 group had an increased PT, measured at 31.9 seconds, which was approximately 3× higher than the baseline levels and an increased APTT, measured at 49.5 seconds, which was approximately 2× the baseline levels and above the average values for all of the other animals in that group on Day 7 post-challenge. This animal had a clinical responsiveness score of '1' from Days 6–9 post-challenge, yet only barely detectable amounts of virus, which were below the lower limit of quantitation for the qRT-PCR viral load assay.

Fibrinogen tends to decrease during severe EBOV infection, which was seen in the two unvaccinated control NHPs at Day 7 post-challenge. In disease states, fibrinogen decreases are likely related to combinations of decreased food consumption, inflammation, and renal insult, if present. On average, fibrinogen increased for all of the NHP in the vaccinated groups after EBOV challenge, without a particular dose-response effect based on dose-level of vaccine. Fibrinogen is considered

an acute phase reactant, and its elevation is indicative of inflammation commonly seen in viral infections, and is possibly part of the anti-viral response in the vaccinated survivor animals.

Antithrombin (AT) is an anticoagulant protein which inactivates thrombin and other coagulation factors. Lower levels, such as those seen in acute EBOV cases and in the two control animals at Day 7 post-challenge, may be indicative of liver failure (where AT is synthesized), presence of disseminated intravascular coagulation, and renal failure. On average, AT did not change for any of the NHP in the vaccinated groups.

Levels of D-dimer, which are products of fibrin clot degradation, increased sharply (>4-fold over baseline) for the two non-vaccinated control animals from Days 5 post-challenge to Day 7 post-challenge, a phenomenon typically seen in severe EBOV infection, and most likely related to the presence of disseminated intravascular coagulation, where there is dysregulation of clotting events. D-dimer increase is frequently seen with prolonged PT and APTT, and decreased fibrinogen and platelet counts, which had been observed for the control animals. D-dimer levels in the vaccinated NHP across all groups were variable, but lower than levels observed in the control animals on Day 7.

SUPPLEMENTARY TABLES AND FIGURES

Table S1. Selection criteria for pseudovirion neutralization assay (PsVNA)

Group	Animal number	Clinical score (maximum)	Leg score (swelling) Days Post-Challenge	Viral load, maximum (genomic equivalents/mL)	Notes/justification for choosing these
Control group (saline)	6-1 (2235)	3	Mild on Days 5 and 7	9.66 $\log_{10}$	Choosing both controls for PsVNA as negative responders
	6-2 (0004)	4	Mild on Day 5; moderate on Day 7	10.07 $\log_{10}$	
Group 1 (highest dose group, 3 × 10^6 pfu)	1-2 (1873)	2	Mild on Day 7; moderate on Day 10	5.49 $\log_{10}$	Sickest animal in Group 1
	1-4 (0554)	0	Mild on Day 7 (only)	Below LLOQ*	Exemplary of healthy animal from Group 1
Group 3 (middle dose group, 3 × 10^4 pfu)	3-1 (0145)	0	Mild on Day 7 (only)	5.01 $\log_{10}$	Clinical score was always zero, even though viral load was quantifiable.
	3-3 (0524)	0	0	None ever detected	All animals in Group 3 never demonstrated a clinical score; this one also demonstrated no leg scoring
Group 5 (lowest dose group, 3 × 10^2 pfu)	5-1 (1648)	0	0	Below LLOQ*	One of healthiest animals in Group 5.
	5-2 (1432)	2	Moderate on Day 7; severe on Days 10 and 14; mild on Days 21 and 28	6.48 $\log_{10}$	This was the sickest animal on study, outside of control animals

* LLOQ is 4.91. LLOQ=Lower limit of quantitation; PsVNA=pseudovirion neutralization assay

Table S2. Antibody titers post-vaccination from the one vaccinated NHP who succumbed on Day 9 post-challenge. Total IgG measured by EBOV GP ELISA and neutralizing antibody titers measured by BSL-2 VSV-pseudotyped PRNT assay are shown for the one NHP in group 3 ($3x10^6$ dose group) who succumbed on Day 9 post-challenge. EU: ELISA Units.

	Day post-vaccination							
	-4	0	7	14	28	35	42 (Day 0 post-challenge)	49 (Day 7 post-challenge)
Anti-EBOV GP IgG titers (EU/mL.)	25.26	0	1.36	279.45	1820.84	1358.63	866.03	38.56
PRNT titers (PRNT60)	NA	<20	126	491	531	481	NA	NA

Table S3. Viral load post-challenge from the one vaccinated NHP who succumbed on Day 9 post-challenge. Viral Load (log10 Genomic Equivalents/mL) after EBOV challenge are shown for the one NHP in group 3 ($3x10^6$ dose group) who succumbed on Day 9 post-challenge.
LOD: Limit of Detection; LOOQ = lower limit of quantitation; GE: Log10 Genomic Equivalent/mL

	Day post-challenge				
	0	3	5	7	10
Viral Load (log10 Genomic Equivalents/mL)	Below LOD, LLOQ	Below LOD, LLOQ	3.08 GE/mL, which is below LLOQ but above LOD	9.73 GE/mL	Had succumbed on Day 9

Supplementary Figure S1. Antibody responses in nonhuman primates after single intramuscular administration of various doses of rVSVΔG-ZEBOV-GP determined using non-validated assays.

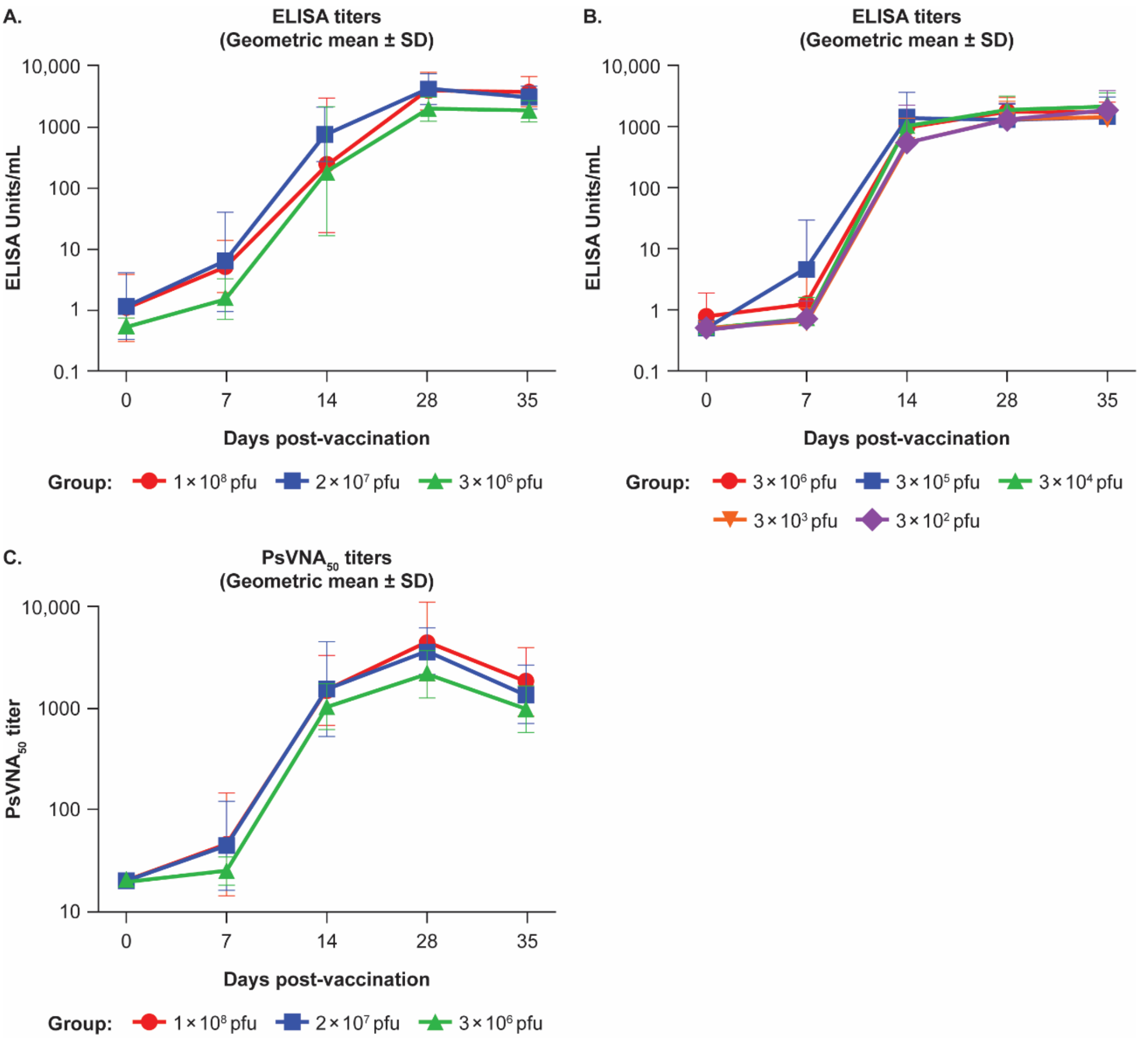

Ebolavirus-glycoprotein (EBOV-GP)–specific immunoglobulin G antibody titers in *A*, Study 1 and *B*, Study 2, and *C*, neutralizing antibody titers in pseudovirion neutralization assay 50% ($PsVNA_{50}$) in Study 1. Three experimental groups (eight monkeys per group) in Study 1 received 1×10^8, 2×10^7, or 3×10^6 plaque-forming unit (pfu) intramuscular (IM) doses of rVSVΔG-ZEBOV-GP. Five experimental groups (four or five monkeys per group) in Study 2 received 3×10^6, 3×10^5, 3×10^4, 3×10^3, or 3×10^2 pfu IM doses of rVSVΔG-ZEBOV-GP.

ELISA, enzyme-linked immunosorbent assay; $PsVNA_{50}$, pseudovirion neutralizing assay 50 titers.

Supplementary Figure S2. Ebolavirus-glycoprotein (EBOV-GP)–specific immunoglobulin G antibody titers in nonhuman primates after single intramuscular administration of various doses of rVSVΔG-ZEBOV-GP in Study 2.

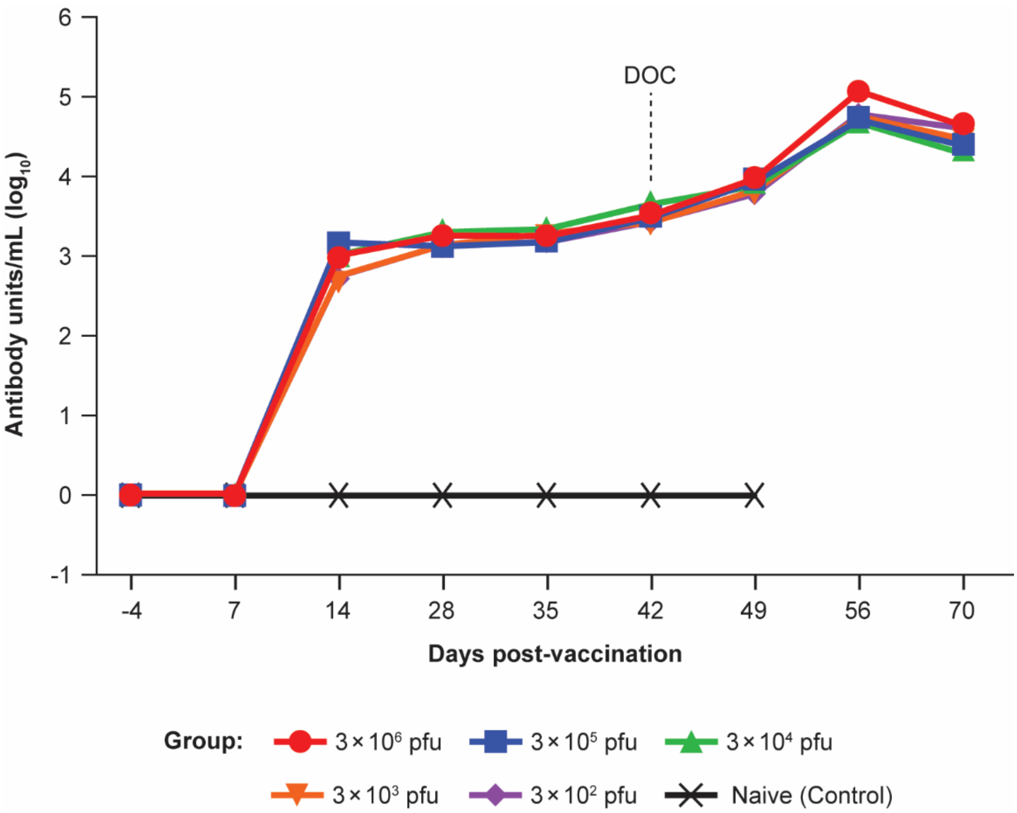

Five experimental groups (four or five monkeys per group) in Study 2 received 3×10^6, 3×10^5, 3×10^4, 3×10^3, or 3×10^2 plaque-forming unit (pfu) intramuscular (IM) doses of rVSVΔG-ZEBOV-GP.

Antibody titers are presented as geometric means

DOC, day of challenge.

Supplementary Figure S3. Neutralizing antibody activity before and after challenge as measured by $PsVNA_{50}$ in Study 2

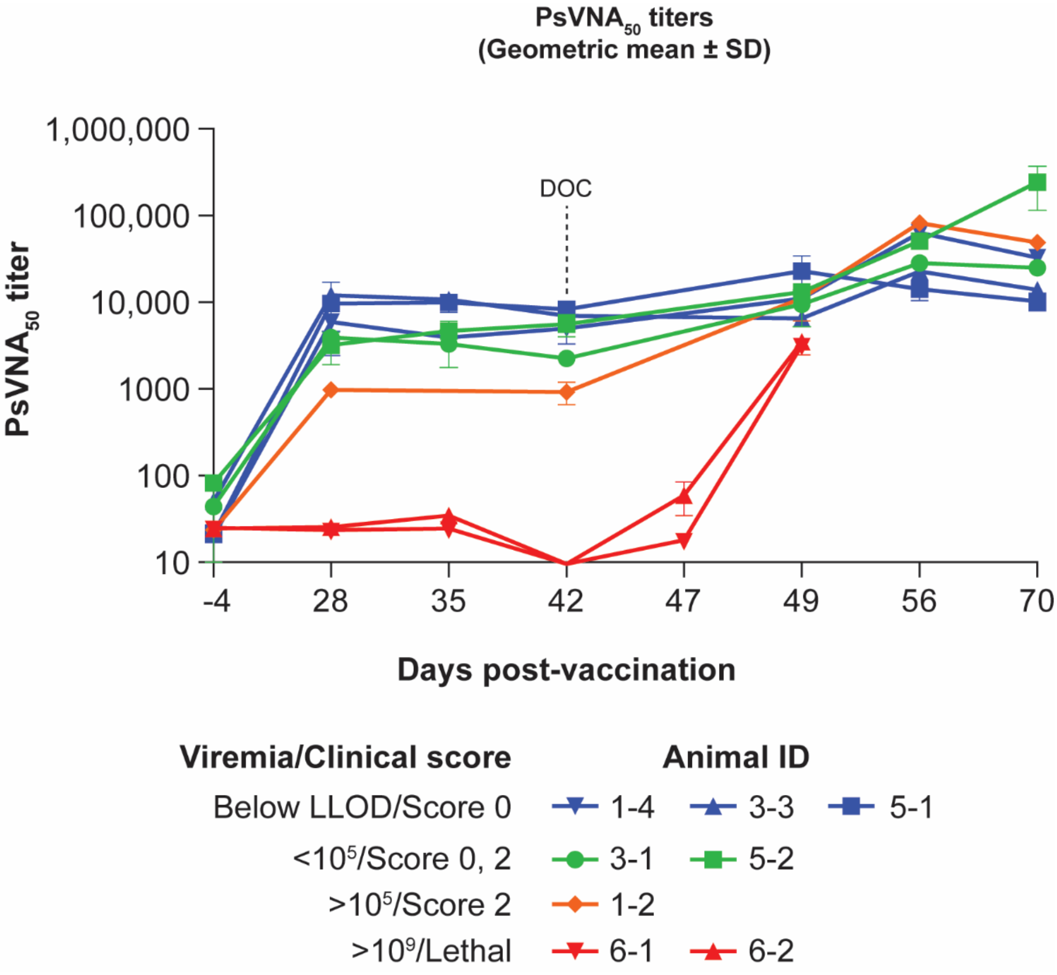

Post-challenge neutralizing antibody responses were further evaluated in 8 animals from Study 2, based on viremia levels and clinical scores after challenge. These included 6 vaccinated animals: 3 with no viremia or clinical signs; 2 with peak viremia <10^5 pfu/mL and clinical scores of 0 and 2; and 1 with peak viremia >10^5 pfu/mL and a clinical score of 2. The other 2 animals (control group) had viremia >10^9 pfu/mL and lethal disease. In the control animals, neutralizing antibody activity was not detected at the time of challenge but rose rapidly, reaching >1000 within 1 week after challenge. In vaccinated animals, neutralizing antibody activity titers were between 1000 and 10,000 at the time of challenge, and then increased at a lower rate after challenge; titers exceeded 10,000 in some immunized animals by Day 56, and 100,000 in one animal by Day 70.

Supplemental References

1. Regules, J.A.; Beigel, J.H.; Paolino, K.M.; Voell, J.; Castellano, A.R.; Hu, Z.; Munoz, P.; Moon, J.; Ruck, R.; Bennet, J.; et al. A Recombinant Vesicular Stomatitis Virus Ebola Vaccine. *N Engl J Med* **2017**, *376*, 330-341, doi:10.1056/NEJMoa1414216.
